# Cost-Effectiveness of an Intervention to Reduce HIV/STI Incidence and Promote Condom Use among Female Sex Workers in the Mexico–US Border Region

**DOI:** 10.1371/journal.pone.0011413

**Published:** 2010-06-30

**Authors:** José L. Burgos, Julia A. Gaebler, Steffanie A. Strathdee, Remedios Lozada, Hugo Staines, Thomas L. Patterson

**Affiliations:** 1 Division of Global Public Health, Department of Medicine, University of California San Diego, La Jolla, California, United States of America; 2 Faculty of Medicine, University of Xochicalco, Tijuana, Mexico; 3 State Public Health Service, Tijuana, Mexico; 4 Faculty of Medicine, Autonomous University of Ciudad Juarez, Ciudad Juarez, Mexico; 5 Department of Psychiatry, University of California San Diego, La Jolla, California, United States of America; Stanford University, United States of America

## Abstract

**Background:**

Previous research demonstrated efficacy of a brief behavioral intervention to reduce incidence of HIV and sexually transmitted infections (STIs) among female sex workers (FSWs) in Tijuana and Ciudad Juarez, Mexico, cities on Mexico's border with the US. We assessed this intervention's cost-effectiveness.

**Methodology and Principal Findings:**

A life-time Markov model was developed to estimate HIV cases prevented, changes in quality-adjusted life expectancy (QALE), and costs per additional quality-adjusted life year gained (QALY), comparing (in US$2,009) no intervention to a once-only and annual intervention. Future costs and health benefits were discounted annually at 3%. Sensitivity analyses evaluated model robustness. We found that for a hypothetical 1,000 FSWs receiving the once-only intervention, there were 33 HIV cases prevented and 5.7 months of QALE gained compared to no intervention. The additional cost per QALY gained was US$183. For FSWs receiving the intervention annually, there were 29 additional HIV cases prevented and 4.5 additional months of QALE compared to the once-only intervention. The additional cost per QALY was US$1,075. When highly active antiretroviral therapy (HAART) was included in the model, the annual intervention strategy resulted in net savings and dominated both once-only and no intervention strategies, and remained robust across extensive sensitivity analyses. Even when considering clinical benefits from HAART, ignoring added costs, the cost per QALY gained remained below three times the Mexican GDP per capita, and below established cost-effectiveness thresholds.

**Conclusions/Significance:**

This brief intervention was shown to be cost-effective among FSWs in two Mexico-US border cities and may have application for FSWs in other resource-limited settings.

**Trial Registration:**

ClinicalTrials.gov NCT00338845

## Introduction

The financial burden of the worldwide HIV epidemic surpasses US$10 billion annually [1], and over US$270 million annually in Mexico [2], where 88% of costs are attributable to highly active antiretroviral therapy (HAART) [3]. In Mexico, HIV was until recently thought to affect almost exclusively men reporting sex with other men (MSM) [4]. However, the proportion of female AIDS cases has increased steadily from 3% in 1986 [5] to more than 27% in 2008, affecting approximately 57,000 Mexican women [6].

Baseline testing of FSWs in an intervention study in Tijuana and Ciudad Juarez in 2006 found a strikingly high prevalence of HIV infection compared to earlier studies of FSWs in Mexico. A 1997 study in Mexico City estimated HIV prevalence among FSWs there at 0.6% [7]; by contrast, HIV prevalence among a large sample of FSWs in Tijuana and Ciudad Juarez in 2006 was 6%, and the same sample showed prevalence of any STI to be 25% [8].

Programs aimed at reducing HIV and STIs among FSWs are thought to be cost-effective since they reduce HIV transmission from high-risk groups to the general population in areas with concentrated epidemics [9], [10]. In Tijuana and Ciudad Juarez, where commercial sex work is quasi-legal and the sex trade is thriving [11], an effective response to the HIV epidemic would include efficacious interventions that have shown reductions in HIV incidence among FSWs in community trials [12]. Considering Mexico's limited resources, which are even further constrained in the current global financial crisis, effective public health interventions need to prove their relative cost-effectiveness before being widely adopted.

*Mujer Segura* (Healthy Woman) is a brief (35-minute) behavioral intervention developed to increase condom negotiation skills and reduce incidence of HIV and STIs among FSWs [11]. Between 2004 and 2006, 709 FSWs in Tijuana and Ciudad Juarez were randomized to receive either *Mujer Segura*, which integrates motivational interviewing and theoretical principles of behavior change, or a time-equivalent didactic presentation critical to HIV and STI prevention [13]. Among FSWs assigned to the intervention, there was a statistically significant increase compared to the control group in reported protected sex acts with clients after six months of follow-up and a 40% reduction in STI incidence (HIV, syphilis, gonorrhea, Chlamydia, or any combination of these). Of note, HIV incidence was zero per 100 person-years in the intervention group versus 2.01 per 100 person-years among FSWs assigned to the control group, as reported previously [13]. We assessed cost-effectiveness of this intervention in reducing HIV/STI incidence among FSWs in the northern Mexican border region, to determine its potential for adoption as a preventive health practice in other resource-limited settings.

## Methods

### Ethics Statement

**Figure 1:**
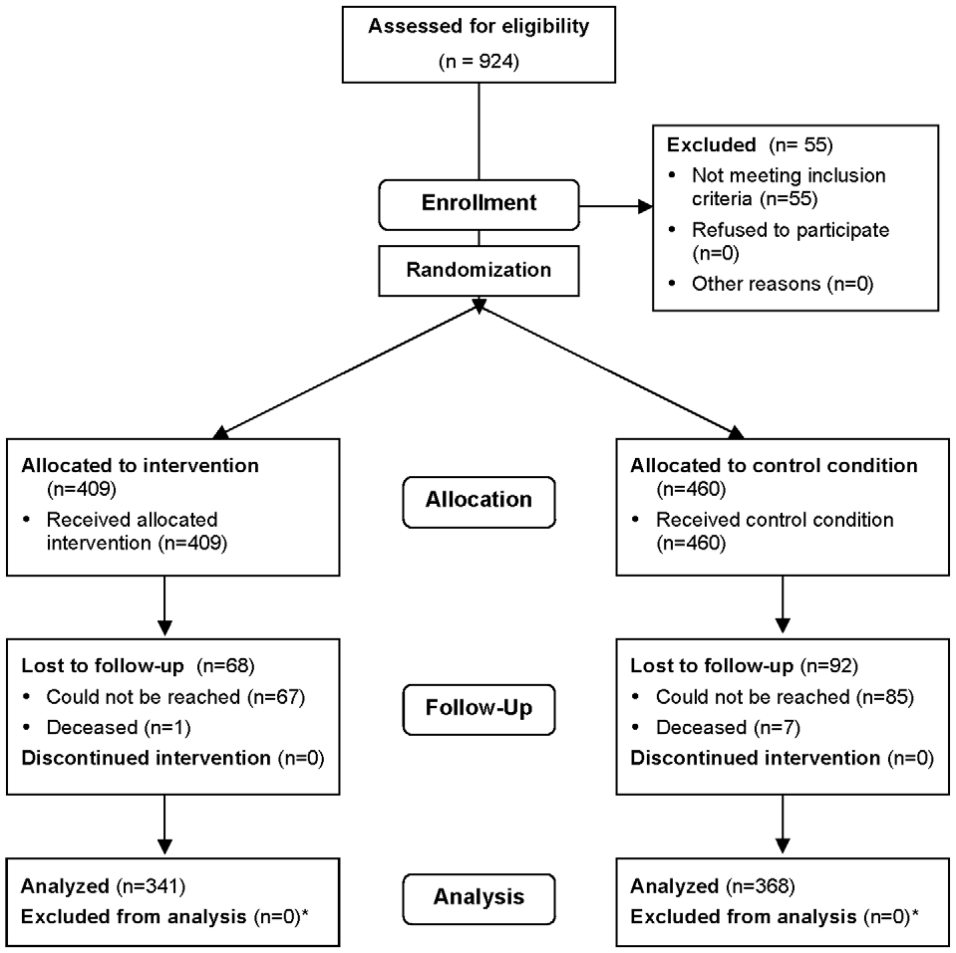
CONSORT flow chart. * All participants who completed follow-up were analyzed for STI incidence. Some participants were lacking follow-up sexual risk data. See Table 2, p. 2054, in Patterson et al. (2008), Am J Public Health 98: 2051–2057 for details.

The intervention study on which these analyses are based was reviewed and approved by the Human Research Protections Program of the University of California, San Diego (Project #051182). The study was registered with ClinicalTrials.gov as Protocol NCT00338845. Written consent was given by the patients for their information received, stored, and used for this research. The protocol for this trial and supporting CONSORT checklist are available as supporting information; see Checklist S1 and Protocol S1. Please note that the page numbers in the Checklist refer to the print version of an article that contains a full description of the methods and results of the trial [13]. The same article is available in full text or PDF from PubMed Central (PMC2633868). The CONSORT Flow Chart for the protocol may be seen in Figure 1.

### Summary

We developed a state-transition Markov model using Treeage Pro Suite software (Treeage Inc., 2009 release 1.0.2, Williamstown, MA, USA) to evaluate cost-effectiveness of the *Mujer Segura* intervention. A Monte Carlo simulation was conducted to calculate clinical benefits (incident HIV infections and quality-adjusted life years or QALYs) and lifetime costs comparing no intervention to the *Mujer Segura* intervention offered once only or annually. Strategies were compared by calculating the incremental cost-effectiveness ratios (ICER), defined as the additional health benefit of an intervention compared with the next least costly strategy [14]. We adopted a government health care payer perspective, the most relevant for health policy decision-making in low- and middle-income countries. Costs are presented in US$2009 according to the 2009 consumer price index and currency exchange rates published by the National Bank of Mexico. Future costs and health benefits were discounted at an annual rate of 3% as recommended by the U.S. Panel on Cost Effectiveness in Health and Medicine [15]. Following the World Health Organization recommendation, we considered an intervention “highly cost-effective” if it was less than one time the per capita gross domestic product (GDP) in Mexico per QALY gained (equivalent to US$ 9,766) and “not cost-effective” if it was greater than three times the per capita GDP per QALY gained (equivalent to US$29,300) [16]. Study protocols were approved by the responsible institutional review boards in the U.S. and Mexico.

### Model structure

**Figure 2:**
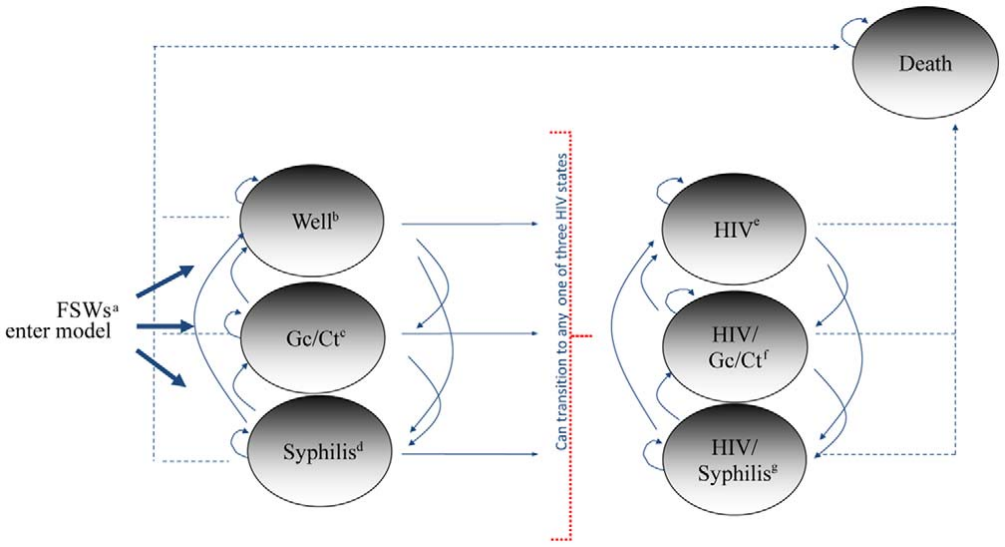
Schematic representation of the Markov model used for the cost effectiveness analysis for the Mujer Segura intervention. a = Female sex workers; b = No HIV or sexually transmitted infections; c = Gonorrhea or Chlamydia trachomatis infection; d = Syphilis infection; e = HIV infection (no other sexually transmitted infections); f = HIV and gonorrhea or Chlamydia co-infection; g = HIV and syphilis co-infection.

Risks of HIV and STI acquisition among FSWs were modeled as a sequence of annual transitions between seven mutually exclusive health states. As shown in Figure 2, FSWs enter the model in one of three mutually exclusive health states free of HIV infection: 1) No HIV or STIs; 2) non-ulcerative STI (i.e., gonorrhea or *Chlamydia trachomatis*); and 3) syphilis infection. After entering the model, FSWs can remain in one of the initial states, or transition to one of four additional states: 4) HIV infection with no concurrent STIs; 5) HIV and a non-ulcerative STI; 6) HIV and syphilis co-infection; and 7) death.

To track HIV progression within the Markov model, we created a variable for CD4+ counts that modified health related quality of life (HRQoL) values, costs, and AIDS-related mortality (Table 1). For example, we assumed that in a person who acquired HIV infection, CD4+ cell counts would decline by 25% during the first year and by 60 cells annually thereafter [17]. For FSWs receiving HAART, we assumed that CD4+ cell counts would increase by 100 cells for the first year on HAART and by 60 cells annually thereafter, until the CD4+ cell count reached 500 [18].

**Table 1:** CD4+ counts and changes in mortality and health-related quality of life values used for the base case analysis.

CD4+ cells^a^	IRR^b^	QALY^c^
>450	1	0.98
400–450	1.41	0.96
300–399	1.45	0.94
200–299	1.66	0.94
100–199	2.59	0.87
50–99	4.63	0.81
0–49	11.63	0.79

a Number of CD4+ cells per micro liter.

b Incidence rate ratio.

c Quality-adjusted life years.

### Monte Carlo simulation

We conducted a Monte Carlo simulation in which individual women with unique characteristics were tracked and followed over their lifetimes [14]. Individual characteristics (initial age, injection drug use, baseline CD4 counts, and years remaining in sex work) of each FSW were randomly assigned using distributions derived from the *Mujer Segura* cohort and other studies [8], [13], [17], [19]. We first conducted the simulation assuming no access to HAART, and subsequently assuming universal access to HAART, initiated according to CD4+ cell count thresholds using data from published reports from Mexico [3], [19]. We reported the number of HIV infections averted and quality-adjusted life expectancy (QALE) comparing no intervention with the *Mujer Segura* intervention offered once only, and the *Mujer Segura* intervention offered annually for a cohort of 1,000 FSWs. Based on previous studies [20], we assumed that FSWs retire from sex work after the age of 57 and stop receiving the *Mujer Segura* behavioral intervention. Additionally, after leaving sex work, the risk for HIV infection and STIs is adjusted according to the age of the general female population in the U.S.-Mexican border region [21].

### Model parameters

Base case clinical and epidemiological variables used to characterize the population of FSWs are presented in Table 2. Variables include the prevalence and incidence of STIs among FSWs [13], annual risk for HIV infection, annual background mortality according to age for Mexican women [22], increased mortality from HIV infection according to CD4 cell counts in the absence of HAART [17], response to HAART [18], years in sex work [20], HAART initiation according to CD4 cell counts thresholds [3], [19], and efficacy of the *Mujer Segura* intervention for reducing HIV/STI incidence among FSWs [13], [23], [24].

**Table 2:** Model base case clinical values and ranges used for sensitivity analyses.

	Variable	Base Case (range)	Source
**Clinical values**	Baseline CD4+ counts^a^	750 (600–1200)	[1], [17]
	Annual CD4+ count decline^a^	60 (37–77)	[17]
	Annual CD4 count increase^a^	100 (60–120)	[18]
**Epidemiological values**	Syphilis incidence^b^	7.75 (3.6–11)	[13]
	Gonorrhea incidence^b^	8 (3.8–12.19)	[13]
	Chlamydia incidence^b^	10.5 (5.51–15)	[13]
	HIV incidence^b^	2.01 (1–3)	[13]
	*Mujer Segura* effectiveness^c^	40% (20–60%)	[13], [23], [24]
	Probability of leaving sex work^b^	21.5 (10–35)	[20]
	Background mortality	Life tables	[22]
**HIV/AIDS medical costs (annual)**	*CD4+ cell counts:*		
	More than 350	$3,745^d^	[3], [25]
	Between 200–349	$4,186^d^	[3], [25]
	Between 100–199	$4,287^d^	[3], [25]
	Less than 100	$5,305^d^	[3], [25]
**Annual probability of initiating HAART** e	*CD4+ cell counts:*		
	More than 350	9%^d^	[3], [19]
	Between 200–349	40%^d^	[3], [19]
	Between 100–199	49%^d^	[3], [19]
	Less than 100	6%^d^	[3], [19]

a cells per microliter.

b Per 100 person years.

c Risk reduction for sexually transmitted infections among female sex workers.

d Probability distribution used for probabilistic sensitivity analysis.

e Highly Active Antiretroviral Therapy.

Base case costs used in the model are summarized in Table 3. Costs were obtained using an ingredients approach [14] for the observed costs per screening (e.g., STI screening, personnel time costs during counseling) during the *Mujer Segura* study. For fixed costs such as space rental and administrative personnel expenditures, we used a step-down approach [14]. Costs associated with treatment of STIs and HIV infections were obtained from the National Center for AIDS Prevention in Mexico (CENSIDA) and published reports from Mexico [3], [25].

**Table 3:** Model base case costs and ranges used for sensitivity analyses.

	Variable	Base Case (range)	Source
**Personnel time costs** a	Counseling sessions	$5.00 (3.50–10.00)	b
	Sample collection	$5.00 (2.50–10.00)	"
**Laboratory tests**	Gonorrhea and Chlamydia	$22.00 (15–30)	"
	Syphilis FTP rapid test	$5.00 (2.50–7.50)	"
	Syphilis RPR^c^	$5.00 (2.50–7.50)	"
	HIV rapid test	$3.50 (3.50–15.00)	"
	HIV confirmatory test^d^	$56.00 (40–65)	"
**Other**	Incentives^e^	$30.00 (15–45)	"
**STI treatment costs**	Azithromycin 1 gr.	$14.00 (10–20)	"
	Ceftriaxone 125 mg.	$7.70 (5–10)	"
	Benzathine penicillin G	$5.00 (3–8)	"
	Three doses for HIV+ FSWs	$15.00 (9–24)	"
**Annual fixed costs**	Space (rent)	$3,600 (1200–7200)	"
	Telephone, internet	$480 (350–600)	"
	Utilities	$900 (600–1,200)	"
	Personnel training	$500 (250–750)	"
	Mileage (outreach, recruiting)	$9,600 (5–10K)	"
	Administrative personnel	$9,000 (8–12K)	"
	Outreach workers	$9,600 (8–10K)	"
	Start up costs	$5,000 (2,500–7,500)	"

a Personnel wages per hour.

b Data obtained from the Mujer Segura accounting records.

c Rapid Plasma Reagin test for syphilis.

d HIV confirmed by immunofluorescence assay.

e Economic incentive given to participants per visit.

### Sensitivity analyses

To account for parameter uncertainty, we conducted one-, two-, and multi-way sensitivity analyses for all input values according to 95% confidence intervals (CI) derived from the *Mujer Segura* cohort study and for the likely range of other inputs according to an extensive literature review, to encompass plausible low and high values (Tables 2–3). We performed a second-order Monte Carlo simulation for a multivariate probabilistic sensitivity analysis using probability distributions from the *Mujer Segura* study and published reports [3], [17], [18], [19], [26]. These methods involved 500 parallel simulations of 10,000 individual random walks each, using different probability distributions to calculate robust confidence intervals. To generate a cost-effectiveness acceptability curve, we conducted a probabilistic sensitivity analysis using Monte Carlo simulation methods modifying the effectiveness of the intervention to reduce HIV incidence using a triangular probability distribution [27] with a range between 0% and 60% with 40% effectiveness as best estimate. Finally, we used multi-way sensitivity analyses to explore combined effects to represent the general female population of Tijuana and Ciudad Juarez.

### Model calibration

We calibrated our model by comparing estimates of life expectancy, HIV incidence, and median survival for women not in sex work to estimates from the 2009 U.S.–Mexico Border Epidemiological Profile [21], the 2005 Centers for Disease Control and Prevention (CDC) HIV/AIDS surveillance report [28], and demographic data from the National Mexican Population Council (CONAPO) [22]. None of these data sources were used to develop the model.

### Role of the funding source

The sponsor of the *Mujer Segura* behavioral intervention study (National Institute of Mental Health) had no role in designing the study, in the collection, analysis, or interpretation of the data, in the writing of this report, or in the decision to submit this paper for publication.

## Results

### Calibration results

Before evaluating the cost-effectiveness of the *Mujer Segura* intervention, we assessed the ability of our model to predict reasonable and valid estimates. Life expectancy obtained by the model for Mexican women not in sex work was close to estimates reported for Mexican women (77.96 years and 77.6 respectively) [22]. HIV incidence for all women was estimated at 10.69 cases per 100,000 person-years, varying between 5.43 and 13.32 per 100,000 for women over 55 years of age and younger women ages 18 to 54 respectively. Overall HIV incidence rates were close to the CDC estimates for Latino women in the U.S. at 11.2 per 100,000 [28]. Similar to findings from other cohorts [29], median survival post-HIV infection predicted by the model was 12 years (interquartile range [IQR]: 9–14) when HAART was not considered in the model, and varied according to the age when HIV infection was acquired, showing increased survival for women infected at younger ages. Considering HAART initiation based on clinical CD4 count thresholds [19], the median survival post-HIV infection was 24 years (IQR: 12–35).

### Base case results

For a hypothetical cohort of 1,000 FSWs from Tijuana and Ciudad Juarez, our analysis indicated that if the *Mujer Segura* intervention were offered once-only, 33 HIV infections would be prevented (95% CI: 30–37), increasing the QALE by 151 (95% CI: 135–171) days per FSW, at a cost of US$183 (95% CI: $164–$206) per QALY, and US$2,370 (95% CI: $2,092–$2,370) to prevent each HIV case (Table 4). If the *Mujer Segura* intervention were offered to FSWs annually, the model suggests an additional 29 (95% CI 26–33) HIV new cases prevented, increasing the QALE by 132 additional days (95%CI: 109–149), at a cost per additional QALY gained of US$1,075 (95%CI: US$931–$1,259), and $13,413 (95%CI: $11,697–$15,077) per HIV case averted.

**Table 4:** Base case results not considering access to HAART for a cohort of 1,000 female sex workers (FSWs).

Strategy	Cost (US$)	Incremental cost (US$)	HIV cases prevented	Incremental cost per HIV case averted (US$)	QALY^a^	Incremental QALY^a^	Incremental cost per QALY^a^ (US$)
No intervention	19,200	—	0	—	21,863	—	—
MS^b^ once	97,400	78,200	33	2,370	22,290	427	183
MS^b^ annually	486,400	389,000	62	13,413	22,652	362	1,075

a Cumulative quality-adjusted life years for a cohort of 1,000 FSWs.

b *Mujer Segura* intervention.

If we consider universal HAART access for clinically eligible FSWs, the intervention offered annually is more effective and becomes less costly compared with the other two scenarios (no intervention or offering the intervention once-only). As seen in Table 5, the intervention offered once increases the QALE by 11 days (95% CI: 8–14) for a net savings of US$2,485 (95% CI: $2,100–$2,758). An additional 9 days (95% CI: 6–13) of QALE are gained for the intervention offered annually, for an additional net savings of US$1,592 (95% CI: $1,260–$1,929). Lifetime HIV care costs averted for each HIV case prevented were estimated at an average of US$60,000 (95% CI: $52,000–$73,000).

**Table 5:** Base case results considering universal access to highly active antiretroviral therapy (HAART) for a cohort of 1,000 female sex workers (FSWs) in Tijuana.

Strategy	Cost (US$)	HIV cases prevented	Incremental cost per HIV case averted	QALY^a^	Incremental cost per QALY^a^
MS^b^ annual	6,190,360	62	Cost-saving	23,580	Net savings
MS^b^ once	7,782,750	33	Dominated	23,553	Dominated
No intervention	10,268,730	—	Dominated	23,523	Dominated

a Cumulative quality-adjusted life years for a cohort of 1,000 FSWs.

b *Mujer Segura* intervention.

### Sensitivity analyses

In two-way sensitivity analysis, we modeled the base case results plus clinical benefits from universal HAART access for clinically-indicated HIV infections (but ignoring the added costs of HAART). We obtained a cost per QALY of $2,436 (95%CI: $2,020–$3,359) for the intervention offered once (compared to no intervention) and $14,136 (95% CI: $10,100–$20,360) for the intervention offered annually (Table 6).

**Table 6:** Base case results considering universal access to HAART, ignoring added costs for antiretroviral medications, for a cohort of 1,000 female sex workers (FSWs).

Strategy	Cost (US$)	HIV cases prevented	Incremental cost per HIV case averted (US$)	QALY^a^	Incremental cost per QALY^a^ (US$)
MS^b^ annual	19,500		—	23,497	
MS^b^ once	97,500	33	2,370	23,529	2,435
No intervention	482,000	62	13,258	23,556	14,136

a Cumulative quality-adjusted life years for a cohort of 1,000 FSWs.

b *Mujer Segura* intervention.

**Figure 3:**
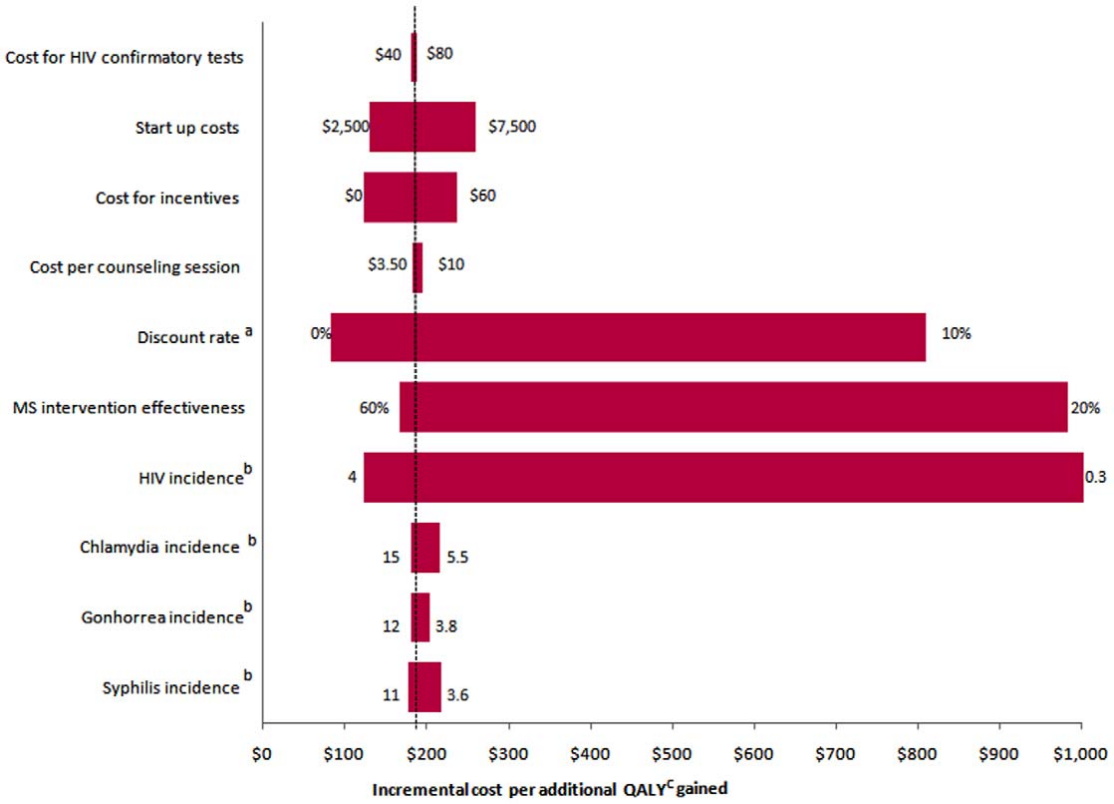
Tornado Diagram showing results of one way sensitivity analyses comparing no intervention to the Mujer Segura intervention offered once only. The vertical dotted line represents the base-case analysis incremental cost per QALY (quality-adjusted life years) gained. The numbers at the end of each bar represents the range over which each of the variables was changed. a = Changes in annual discount rate used for costs and health benefits; b = Incidence is presented per 100 person-years; c = quality adjusted life years.

**Figure 4:**
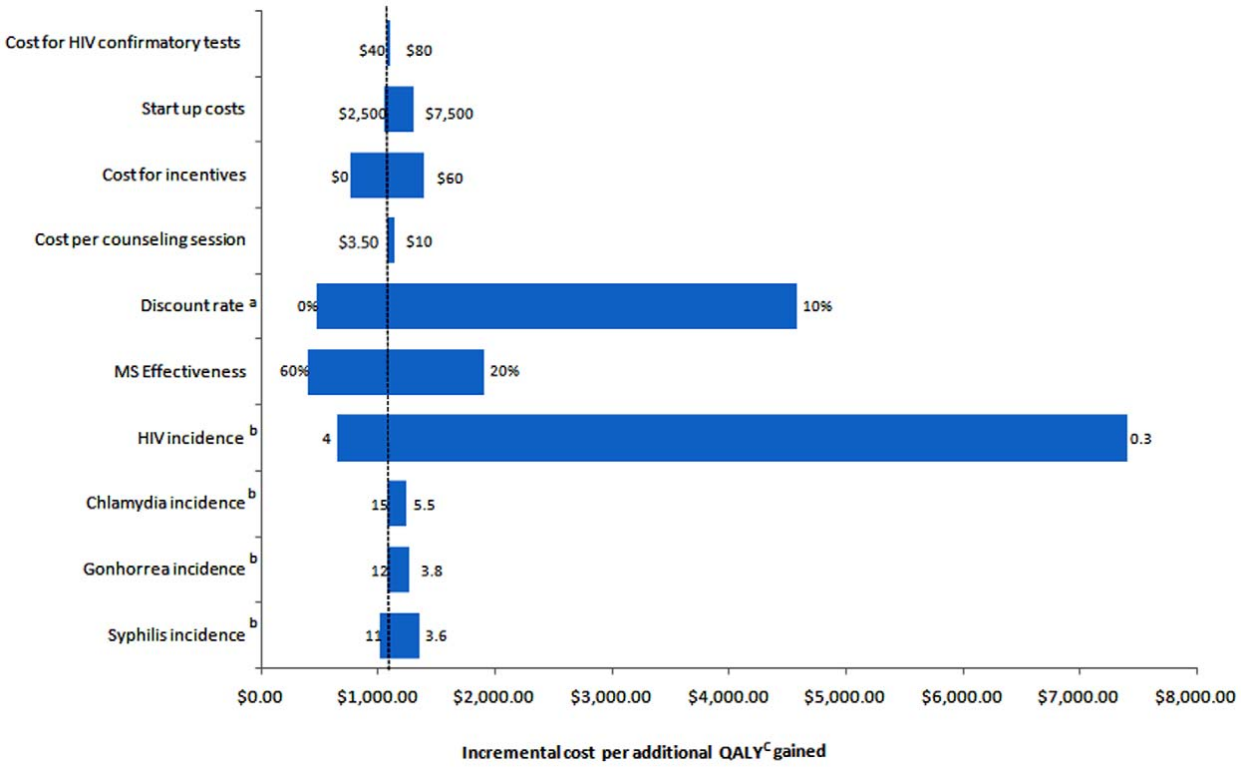
Tornado Diagram showing results of one way sensitivity analyses comparing the Mujer Segura intervention offered once only and annually. The vertical dotted line represents the base-case analysis incremental cost per QALY (quality-adjusted life years) gained. The numbers at the end of each bar represents the range over which each of the variables was changed. a = Changes in annual discount rate used for costs and health benefits; b = Incidence is presented per 100 person-years; c = quality adjusted life years.

In one-way sensitivity analyses, results were sensitive to changes in HIV incidence. Compared to no intervention, for the once-only intervention, when HIV incidence increases to 4 per 100 person years, the cost per QALY becomes more favorable at US$122; when incidence falls to 0.3 per 100 person years, the cost per QALY gained increases to US$1,202. For the intervention offered annually, the cost per QALY ranged from US$600 to $7,409 compared to the once-only intervention, for an HIV incidence of 4 and 0.3 per 100 person years respectively (Figures 3 and 4).

**Figure 5:**
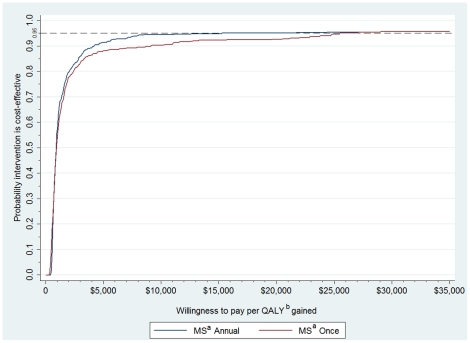
Cost-effectiveness acceptability curve for the Mujer Segura intervention offered once or annually. Results of the probabilistic sensitivity analysis, represented as a cost-effectiveness acceptability curve. The cost per QALY (quality adjusted life years) gained from the Mujer Segura intervention is plotted on the x-axis, and the probability that the intervention is cost-effective across these values is plotted on the y-axis. The Mujer Segura intervention offered once or annually resulted in a cost-effectiveness below a willingness to pay treshold of three times the GDP in Mexico per QALY gained (equivalent to US$29,300). a = Mujer Segura behavioral intervention; b = Quality adjusted life year.

The cost effectiveness acceptability curve (Figure 5) generated from the probabilistic sensitivity analysis indicated that there was a greater than 95% probability of a cost per QALY gained less than US$25,499 for the interventon offered once and US$15,200 for the intervention offered annually.

When the *Mujer Segura* intervention was targeted to the general female population in Tijuana or Ciudad Juarez with HIV incidence of 10 per 100,000 person-years and STI incidence of 10, 89 and 386 per 100,000 person-years for syphilis, gonorrhea and Chlamydia respectively [21], the intervention is no longer cost-effective, with a cost per QALY of US$98,000 (95%CI: $43,000–$202,000), greater than three times the GDP in Mexico.

## Discussion

Public health interventions that can effectively reduce HIV risk behaviors among individuals at high risk, such as FSWs, are of critical importance for successful HIV prevention programs [9], especially in resource-limited countries such as Mexico, where there is a dynamic, rapidly evolving HIV epidemic on the country's northern border with the U.S. [30]. Our study shows that the *Mujer Segura* behavioral intervention targeted to FSWs annually is highly cost-effective in Tijuana and Ciudad Juarez, and is cost-saving when averted HAART costs due to the intervention are considered. Furthermore, considering Mexico's stage of the epidemic, individually-based behavioral interventions targeted to bridging populations like FSWs could have a high public health impact at relatively low costs [12]. Even when we considered the clinical health benefits from HAART and ignored the added costs, the cost per QALY gained from this intervention remained below three times the GDP per capita in Mexico and well below the accepted cost-effectiveness threshold for Mexico according to WHO guidelines [16].

The sensitivity analyses we conducted suggested that the cost-effectiveness of expanding the *Mujer Segura* intervention to other populations depends on factors driving HIV incidence. Specifically, our model suggests that it may not be cost-effective to target the *Mujer Segura* intervention to the general female population (i.e., to women with low HIV and STI risk in Tijuana and Ciudad Juarez). This finding is supported by health economists, who suggest that the target population for an intervention should vary according to the stage of the HIV epidemic [31]. For example, in areas where overall HIV prevalence is either low or concentrated in specific populations (such as in the Mexican context), interventions should be prioritized for high-risk groups such as FSWs and MSM. As HIV prevalence becomes generalized, however, interventions should increasingly focus on the general population [10]. Attention must also be given to differential effectiveness of similar interventions among various subpopulations. For example, our work suggests that FSWs who also inject drugs may not benefit as much from our intervention [20], and studies of cost-effectiveness are highly sensitive to contextual variations in local epidemics. This emphasizes the need to select interventions based on background characteristics of the specific population as well as the stage of the epidemic [12].

Unlike other HIV-preventive interventions, the *Mujer Segura* intervention showed efficacy beyond intermediate endpoints (e.g., reductions in reported unprotected sex acts) in reducing HIV incidence among FSWs in community trials in two Mexican border cities [26]. Our individually-administered intervention was both efficacious and cost-effective in high-risk FSWs, but alternative approaches may also be cost-effective in HIV prevention among FSWs in different cultures and epidemic stages. Sweat and colleagues [32] examined two FSW interventions in the Dominican Republic, which is experiencing a more generalized HIV epidemic. The first intervention focused on environmental factors (community mobilization, promotional media, and interpersonal communication and counseling), and a second focused on both environmental and structural factors (such as imposing financial sanctions on sex establishment owners who failed to follow the intervention). While both interventions resulted in cost-effective outcomes, the intervention that included policy regulation was substantially more cost-effective. Alternative prevention approaches shown to be cost-effective in other populations include structural interventions, female condom distribution, HIV rapid tests, and voluntary HIV counseling and testing [33].

Underscoring the importance of evaluating the cost of preventing new cases of HIV infection, the U.S. National Institutes of Health funded the “Prevent AIDS: Network for Cost-Effectiveness Analysis (PANCEA)” study. Information was collected from 228 programs in five low- and middle-income countries (including Mexico) in order to evaluate the relationship between program efficiency (measured as the unit cost) and scale of the program (measured in number of services delivered) [34]. The study found that prevention costs decrease with the scale of the intervention [34]. These findings imply that HIV prevention programs across the globe will become less costly as they continue to grow, which argues in favor of implementation of highly cost-effective behavioral intervention programs like *Mujer Segura*.

This cost-effectiveness analysis has a number of limitations. First, data used to evaluate intervention efficacy were limited to six months of follow-up, raising concerns about the durability of the response beyond this period. Therefore, conservative estimates of efficacy over 12 months were based on published reports from trials among high-risk methamphetamine users in the U.S. using a similar brief behavioral intervention [11]. Second, FSWs in Tijuana and Ciudad Juarez participating in the *Mujer Segura* trial represented a sample at high risk for HIV and STI infection; thus, results from our analysis might not be generalizable to other settings. Although multivariate sensitivity analyses were used to present reliable confidence intervals, parameters used to model HIV progression were obtained from published reports from other populations in the US and Africa.

This brief behavioral intervention reduced incident STI and HIV cases by 40%. Results from the present analyses suggest that the intervention could have a significant public health impact among FSWs in Tijuana and Ciudad Juarez, well below accepted thresholds for cost-effectiveness in Mexico under all plausible assumptions. Unfortunately, behavioral interventions continue to be largely ignored in prevention strategies. While secondary programs such as “seek and treat” have gained popularity and will no doubt continue to play a major role in slowing the HIV epidemic [35], our analysis demonstrates a way in which limited public health resources can be optimized even after taking HAART expenditures into account. Mexican health authorities have expressed interest in scaling up the *Mujer Segura i*ntervention throughout Mexico in collaboration with grass-roots, community-based organizations. We therefore recommend that resources be allocated to identifying both barriers and facilitators to large-scale implementation of this and similar cost-effective behavioral interventions.

## Supporting Information

Checklist S1 CONSORT Checklist
(0.19 MB DOC)

Protocol S1 Trial Protocol
(0.19 MB DOC)
