# Supplementary material for: Cost-Effectiveness of an Intervention to Reduce HIV/STI Incidence and Promote Condom Use among Female Sex Workers in the Mexico–US Border Region
Source: PLoS One. 2010 Jun 30;5(6):e11413. doi: 10.1371/journal.pone.0011413 (PMC2894974; doi:10.1371/journal.pone.0011413)
Supplement: Protocol S1 — Trial Protocol (0.19 MB DOC) [file pone.0011413.s002.doc]

| UCSD Human Research Protections Program **New Biomedical Application**  **RESEARCH PLAN**  Version Date 3/30/2004  (Enter text in the **white space areas** below each numbered heading bar. **Expand the size of table cells as needed** – to multiple pages if needed.  See accompanying Instructions for explanation of headings and information to be provided) |
| --- |
| 1. **PROJECT TITLE** |

| Safer Sex Intervention for Female Sex Workers in Mexico |
| --- |

| **2. PRINCIPAL INVESTIGATOR** |
| --- |

| Thomas L. Patterson, Ph.D. |
| --- |

| **3. FACILITIES** |
| --- |

| This study is being carried out in four cities in Mexico which border on the U.S. The proposed intervention will be delivered in clinics, or nearby facilities, which provide medical examinations for Female Sex Workers (FSWs) in each of these cities. |
| --- |

| **4. ESTIMATED DURATION OF THE STUDY** |
| --- |

| Five Years (2003–2008) |
| --- |

| **5.** **SPECIFIC AIMS** |
| --- |

| Female sex workers (FSWs) are overrepresented in the number of reported cases of AIDS and other STDS in Mexico; yet, efforts to change high risk sexual behavior in this population have been limited. The dramatic increase in HIV/STDS over the past decade (particularly in Mexican cities along the U.S. border)^1^ has placed a strain on already limited health care resources; thus, funding for prevention programs that target FSWs has been virtually non-existent. In December 2001, the San Diego/Tijuana-based research team completed a year-long pilot study that assessed the feasibility of implementing a culturally-sensitive sexual risk reduction counseling program for female sex workers (FSWs) in Tijuana, Mexico. Findings suggested that our sexual risk reduction counseling program for Mexican FSWs was a success, and also suggested the feasibility of implementing our HIV/STD prevention program in other border cities throughout Mexico. The overarching objective of the proposed study is to test the efficacy of our sexual risk reduction counseling program -- PROYECTO COMPARTE SEXO MAS SEGURO (Share Safer Sex Program) -- with a broad-based population of FSWs who reside in Mexican cities along the U.S.-Mexico border. This 5-year study is utilizing a four-city design; there will be two pairs of cities ranging in size from 159,000 to 1.2 million. The four border cities that will participate in this study are Tijuana, Ciudad Juarez, Nuevo Laredo, and Matamoros. These four cities cover 82.5% of the Mexican border population^2^. These cities were chosen because they all have a thriving sex trade industry and are considered “sexual tourist” destinations that attract a large number of clients via U.S border cities. Because of the thriving sex trade industry, border cities have some of the highest rates of HIV/STD cases in Mexico^1^. |
| --- |

| **6. BACKGROUND AND SIGNIFICANCE** |
| --- |

| The U.S. shares a 2,000 mile border with Mexico. This stretch of land encompasses four U.S. southern border states (Arizona, California, New Mexico, and Texas) and six Mexican northern border states (Baja California, Sonora, Chihuahua, Coahuila, Nuevo Leon, Tamaulipas). The border region has a population of over 5 million^2^. Border cities in Mexico are economically depressed by U.S. standards, and commercial sex work is considered quasi-legal. The permissive environment in Mexican border cities attracts large numbers of “sexual tourists” from Mexico, the U.S. and other foreign countries. As a consequence, border cities have some of the highest rate of AIDS cases in Mexico. For example, in 1997, there were 62 AIDS cases per 100,000 residents in Tijuana. This is significantly higher than the 29.9 cases per 100,000 reported for Mexico as a whole^5^. In a recent publication released by the Mexican government’s agency for public-health responses to HIV/AIDS and STDs (CENSIDA), it was reported that over 20% of AIDS cases reported in Mexico came from the six Mexican border states^1^. Another factor besides sex tourism that contributes to the high rates of HIV/STDs in Mexican border cities is the seasonal migration of workers to and from the United States (mostly California). In Mexico, epidemiological surveillance of at risk populations is lacking; the most recent comprehensive data from Tijuana on HIV/STD seroprevalence were gathered in 1990. At that time, the prevalence of Hepatitis B among female prostitutes in Tijuana who did not use drugs was 8.2 percent, compared to 2.2 percent among female control subjects. Female sex workers (FSWs) also had higher rates of positive tests for syphilis as compared to controls^6^. The prevalence of HIV-1 in a sample of 415 FSWs was found to be 0.5 percent^7^, and the prevalence of HTLV-1/2 was 2 percent among FSWs^8^. In a 1995 study, Valdespino reported the following rates of sexually transmitted diseases among female sex workers in Mexico: active syphilis (23.7%), 1odríguez (12.8%), active gonorrhea (11.6%), and hepatitis B antibodies (5.1%)^9^. Recent discussions with health officials in Tijuana and other border cities suggest that the prevalence of STDs and HIV has increased dramatically over the past decade. A recent report suggested a 10% prevalence of HIV among FSWs in Tijuana. Sex with FSWs has been identified as a primary risk factor associated with exposure to HIV and other STDS, particularly the hepatitis C virus and the herpes simplex virus type 1 and 2^10^. These findings suggest that FSWs who perform commercial sex work in Mexican border cities engage in sexual risk behaviors that place them and their clients at significant risk for contracting HIV/STDs. |
| --- |

| **7. PROGRESS REPORT/PRELIMINARY STUDIES** |
| --- |

| Data collection from the first phase of the study (involving Tijuana and Ciudad Juárez) is now complete. A total of 900 female sex workers (FSWs) 18 or older who reported having unprotected sex with at least one client within the previous month were recruited, 450 in Tijuana and 450 in Ciudad Juarez, Mexico. After a baseline assessment, participants were randomly assigned to either: (1) Proyecto Comparte Sexo Mas Seguro (“Share Safer Sex”), a theory-based counseling intervention to increase the use of condoms; or (2) time-equivalent voluntary HIV counseling and testing. FSWs in the intervention and comparison conditions showed no significant differences in baseline demographic characteristics, sexual risk behaviors, or HIV serostatus, which indicates that randomization was successful. A total of 171 (19%) participants were lost to followup, which is a low attrition rate for a study of this kind. Data are currently being analyzed to determine the efficacy of the Comparte Sexo Más Seguro program as compared to the time-equivalent counseling program. As reported last year,^10a^ substantial differences were found in certain psychosocial, disease-prevalence, and drug-use variables between the Tijuana and Juárez cohorts, which could provide the basis for a future tailoring of the subject intervention to local conditions. That article, in turn, formed the basis for a poster presentation at the 2006 International AIDS Conference in Toronto.^10b^ Meanwhile, members of our research team have written additional studies of the data obtained in the first phase. Dr. de la Torre authored an article examining the impact of price and workplace on female sex worker condom use in Tijuana and Ciudad Juarez.^10c^ Also at the 2006 International AIDS Conference in Toronto, a student trainee presented preliminary results of her study of the relationship between workplace type (e.g., brothel vs. street) and sexual risk behavior among FSWs in Tijuana and Ciudad Juárez.^10d^  The second phase of the study, to be carried out in Nuevo Leon and Matamoros in Tamaulipas State, is well under way. All staff have been recruited and trained, and 40 new participants (of a projected total of 900 for these two sites) have been recruited.  There are no site monitoring reports, audits or other reviews to report since the last IRB approval in October, 2005. |
| --- |

| **8. RESEARCH DESIGN AND METHODS** |
| --- |

| This four-city study uses a pre-test, post-test, random assignment, In each treatment city, 450 female sex workers who have had unprotected sex with a client at least once during the previous four weeks will participate in baseline assessment; then randomly assigned to either Condition A (Share Safer Sex counseling session) or Condition B (the CDC’s safer sex program); and finally a six-month followup assessment. Concurrently, another 450 FSWs the second city will participate in the same programs. As noted above, we have completed our data-collection work in Tijuana and Ciudad Juarez, and we have moved on to the second pair of cities (i.e., Nuevo Laredo and Matamoros). As in the first phase of the study, randomization to condition A or B will be performed using a fixed, computer generated randomization scheme to ensure approximately equal allocation to each study condition. In order to reduce potential contamination between the two counseling conditions, participants will be randomly blocked by condition weekly.  Condition A: Within each of the cities, 225 FSWs, a random selection of the 450 FSWs in each city, who meet eligibility criteria and complete a baseline 60-90 minute sexual risk appraisal interview, will participate in the 45-minute Share Safer Sex counseling session. This one on one intervention utilizes motivational interviewing techniques and principles of SCT/TRA to address the context of unsafe sex and condom use with clients (see more detailed description of interventions below). A total of 900 FSWs will receive Share Safer Sex counseling in four cities (225/city).  Condition B: Within each of the cities, 225 FSWs, a random selection of the 450 FSWs in each city, who meet eligibility criteria and complete a baseline 60-90 minute sexual risk appraisal interview, will participate in the 45-minute, one on one, CDC-based “standard counseling” session that offers a personal risk assessment and strategies for reducing personal risk. A total of 900 FSWs will receive the CDC program in the four cities (225/city).  Description of Four Study Cities. Each of the four participating study cities has a large number of female sex workers, ranging from 2,200 in Nuevo Laredo to 6,500 in Tijuana and Ciudad Juarez^11^. The majority of the sex workers receive monthly medical care at the municipal clinic in their city. The large number of sex workers in each city, will help to ensure the successful recruitment of 450 FSWs in each treatment and comparison city. Apart from the pilot study that was conducted by our research team in Tijuana, none of the participating cities has in the past or present offered sexual risk reduction counseling programs for FSWs. Below, we provide a brief description of each study city, and indicate the estimated number of female sex workers. We begin with a description of the border city of Tijuana, Mexico. Tijuana is the largest port of entry to the U.S, and the second largest city along the US-Mexico Border (i.e., 1,212,232 residents). In 1999, there were 18.7 million crossings between Tijuana and San Diego, CA^12^. The main industry in Tijuana is maquiladora assembly plants. Tourism is the second largest industry in Tijuana. Main attractions include bullfights, nightclubs, and bars. Health officials in Tijuana estimate that there are approximately 6,500 female sex workers in this border city^11^. The largest Mexican border city is Ciudad Juarez. Ciudad Juarez is the also largest city in the state of Chihuahua. It has a population of 1,217,818 and is the port of entry from the U.S. city of El Paso, Texas. In 1999, there were 20.9 million border crossings at this location^12^. The main industry in Cuidad Juarez is maquiladora assembly plants. It is estimated that there 6,500 female sex workers in the city of Ciudad Juarez^11^. Nuevo Laredo is the forth largest border city in Mexico, with a population of approximately 750,000. It shares a border with Laredo Texas It is the main gateway to the U.S. for the trucking industry with approximately 80% of all trucks entering the U.S. from Mexico passing through this city.. In addition to trucking, the main industries are agriculture and maquiladora assembly plants. Mexicali shares a border with the U.S. city of Calexico/El Centro. Popular tourist attractions include bullfights, nightclubs, and bars. It is estimated that there are 3,000 female sex workers in the city of Nuevo Laredo. Nuevo Laredo and Matamoros are similarly sized border cities. Matamoros has 416,428 residents is the second largest city in the State of Tamaulipas. Matamoros shares a border with Brownsville, TX. Matamoros is the main port of entry for the U.S. population from Texas and New Mexico. In 1999, there were 7.1 million border crossings at this location^12^. The main industries in Matamoros include agriculture, maquiladoras, and tourism. It is estimated that there are 2,200 female sex workers in the city of Matamoros^11^.  Description of Study Sites: In the proposed study, all interviews and counseling sessions will be conducted at either Government-operated Municipal Medical Clinics, NGOs who specialize in FSWs, or clinics set up by our staff. All four participating cities have clinics located downtown, and within walking distance for the majority of FSWs where treatment referrals will be made if necessary. Each clinic is equipped with an OBGYN examination room, and several additional exam rooms. Women are seen on a first-come, first-serve basis. They register at a desk at the entrance and await their turn. Although some of these clinics are funded by the local municipal governments, the women are expected to pay $40 US for the cost of their medical visit and lab work. Lab work is performed by clinic-affiliated laboratories. Because the municipal clinics are directly responsible for STD/HIV surveillance, they provide clinical services to active sex workers, both male and female. Typically, municipal clinics are staffed with three physicians (including an OBGYN), and three clinical nurses. A social worker and psychologist are also available to provide medical and social services to FSWs. In order to receive or renew a work permit, FSWs are required to undergo clinical examination at a municipal clinic. Only municipal clinics have government accreditation to issue work permits to FSWs. In order to enrich our sample we also recruit women from bars and other venues who are not seeking work permit. These women are brought to the study clinic for interviews and exams.  Recruitment Procedure: Recruitment is conducted at municipal clinics, NGOs, or using street outreach in each participating city. Recruitment of FSWs at the Municipal Clinic in Tijuana (SMM), and street outreach was highly successful; thus, this general approach to recruitment will be utilized in all clinics in the proposed study. Other methods of outreach are standard in the field. The key component to gaining full access to the women was to “cultivate” relationships with the secretaries, receptionists, and nurses who have great influence on the dynamics of SMM. The research staff was very respectful and valued their opinion on recruitment issues. Women were approached after they signed up for their appointment and offered participation. The refusal rate was very low (i.e., 2.5%).  Study Procedure: Recruitment of FSWs into this study will utilize a lagged design. In each treatment city, we will recruit, counsel, and conduct followup interviews with approximately 450 FSWs over a 30-month period. (There will be two 30-month waves of data collection to coincide with our two pairs of cities). Concurrently, 450 FSWs in each city will participate in baseline assessment, counseling, and six-month followup evaluation. FSWs will be interviewed alone by clinic staff who are the same gender. The staff in each treatment city will be comprised of one female interviewer and one counselor both of whom will have had prior experience working at the designated site. In our pilot work, we found that familiarity with the interviewer and counseling staff enhanced the candidness of FSWs responses. Each recruitment site will also be staffed by a site manager and clinical coordinator.  Description Of Assessment Procedure. All assessments will take place in the clinic. Participants will be interviewed face-to-face in privacy by our staff. Because of low reading level, all materials will be administered verbally. The assessment battery requires approximately 60 to 90 minutes to complete.  Study Eligibility: Inclusion criteria. Participants must be women (at least 18 years old) who self-identify as a “female sex worker” and report having had unprotected vaginal, oral, or anal sex with a client at least once during the previous four weeks. Exclusion criteria include: (1) consistent use of condoms/dental dam for vaginal, oral and anal sex with all clients during the previous two weeks; and (2) employed as a sex worker for less than 4 weeks (i.e., to match our 4-week period of recall); (3) under 18 years of age. The latter exclusion criterion was imposed because it is illegal for women under the age of 18 to engage in prostitution in Mexico. Accordingly, FSWs under the age of 18 would not attend the municipal clinic for health care and therefore would not be recruitable. We considered allowing males who identified as FSWs into this study; however, in our pilot study less than 2% of clients at the Municipal Clinic were men who identified at FSWs. In the proposed study, we anticipate an insufficient number of males who identify as FSWs to make their data usable; hence, they have been excluded from study participation. Also, a study of male sex workers would require different recruitment strategies and a targeted intervention to reflect the sexual behaviors and sexual milieus of this population.  FSWs who report intravenous drug use will be included in our study. While our pilot data indicate a low frequency of intravenous drug use (IDU) among FSWs in Tijuana, which is consistent with other studies of Mexican FSWs, we want our sample to be inclusive. Our intervention counseling does not address injection drug use; however, FSWs who report that they have ever injected drugs or vitamins will be provided with written materials (Spanish) on safer injection practices. For those who are illiterate, the written materials provide detailed graphic depictions of how to clean a needle. Also, FSWs who do not have work permits will be allowed to participate in this study.  Definitions of safer sex. We define sexual risk behavior as unprotected insertive or receptive sex (vaginal, oral, or anal). Our definition includes unprotected oral sex. The U.S. Surgeon General’s report states that “getting semen, vaginal secretions, or blood from an infected person into your mouth puts you at risk of HIV infection”^13^. Although the risk of HIV infection from oral sex is lower than from VA sex, sores or cuts anywhere in the mouth make oral sex more risky^13^. Safer sex practices are defined as the use of condoms, or the practice of non-penetrative sex (e.g., manual sex). DiClemente and Peterson^14^ argue that most adults will fail to adopt sexual abstinence as a prevention strategy, thus, appropriate and consistent use of condoms is the most effective method for reducing HIV/STD exposure. In this study, we promote condom use and non-penetrative sex as the best HIV/STD prevention methods. Also, we promote harm reduction strategies, which have lower levels of risk (e.g., when engaging in oral sex have client ejaculate outside of mouth).  Training Procedure: Interviewers and counselors from all sites have received a two-day training led by the UC Davis training team. This program includes the following modules: (1) an overview of the proposed research project, including an overview of data on the sexual health of women in Mexico;(2) research ethics and treatment of human subjects; (3) an overview of potential population based cultural biases and ethnographic research methods, (4) appropriate interview methods, (5) cultural sensitivity as it impacts sexworkers in Mexico, (6) the “culture” of prostitution in Mexico, (7) public health issues impacting sex workers in Mexico, (7) identifying and handling co-morbidity issues that may surface during interviewing and counseling sexworkers , (8) issues in prevention; (9) appropriate intervention strategies; (10) safety considerations for interviewers and counselors, and (11) key points in addressing self-esteem issues with sexworkers. Self-assessment by the participants resulted in an overall high assessment of the training workshop. All training was conducted in Spanish and included a Spanish-language handbook with key points covered during the training. Counselor training procedures were adapted from the earlier pilot study and will continue to be adhered toin the remaining two study cities.  Subject Accrual and Attrition: At the end of the five-year study period, we expect to have evaluated a total of 1,800 FSWs. The first wave of the study, which recruited 900 FSWs in Tijuana and Ciudad Juárez, took about 30 months to complete, and we expect the second phase (in Matamoros and Nuevo Laredo) to be similar. Staff in each city will recruit, assess, and counsel 450 FSWs (for a total of 900), and the same research procedure will be followed.  The first wave of the study showed a retention rate of 81%, which was approximately what we expected based on the rate for the pilot study in Tijuana, which was 79%.  Research Incentives. In order to provide an incentive for participation, we propose to continue paying $30.00 per visit. We will also continue to pay for STD/HIV laboratory tests ($40.00 U.S. per visit). All participants, whether in the treatment or comparison group, will receive approximately 100 condoms and some sexual lubricant. These incentives are modest enough to be non-coercive but substantial enough to encourage complete participation. The laboratory tests serve two purposes: they offer valuable health information to all participants, and they provide objective outcome data for the study.  Notification of Test Results. Laboratory test results will be given to the FSWs by Clinic Staff. When positive test results are obtained, standard notification, counseling, and treatment will be provided by the clinic.  Limitation of Study Design. There are at least two potential problems associated with our study design. The first is the possibility of non-random attrition. It is possible that women who are infected with an STD/HIV will be less likely to come back to the clinic since they may lose their health card/work permit. We argue that this is unlikely since there are few clinics available, and we will pay for clinic visits and laboratory tests. The second potential problem is that randomization of cities could lead to problems such as secular trends affecting our results. This was an issue that was vigorously debated by our research team; in the final analysis, the problems with contamination within single clinics were felt to override the potential problems with our proposed design. Our teams in each of the comparison cities will monitor changes in the clinics and changes in health policy that might affect our results. Stigmatization toward FSWs. At the simplest level, we need to beware of negative labeling that might lead to discrimination against participants. We will protect the confidentiality of our participants to the full extent allowed by U.S. law, including an NIH Certificate of Confidentiality. On another level, stigmatization could impede our efforts to change that most personal of all behaviors, i.e., sexual behavior. Our counselors will be non-judgmental; they will respect the participant’s individuality and take into account the emotions and personal values associated with sexual behaviors; and they will empower participants with the tools and skills they need to effect behavior change. DESCRIPTION OF PROYECTO COMPARTE SEXO MAS SEGURO: The development of intervention materials relied heavily upon our pilot work conducted in Tijuana, Mexico. It also borrowed from our experience conducting sexual risk reduction interventions in the U.S. In the proposed study, clinic-based health care staff are trained to deliver a sexual risk reduction intervention to FSWs. The content of our counseling sessions is tailored to the needs, values, beliefs, and behaviors of our target population. Our protocol takes into account gender differences in counseling issues, with special consideration given to the ecological validity of our intervention for Latinas. It combines principles of motivational interviewing, social cognitive theory, and the theory of reasoned action. To this end, the counselor asks questions about the participant’s condom use with clients, including her perceived need to change, perceived possibility of change, self-efficacy for change, and stated intentions to change. The counselor works with the participant to increase her awareness of current unsafe behaviors and associated risks (e.g., HIV, STDs, pregnancy). Motivational interviewing techniques (e.g., key questions, reflective listening, summarization, affirmation) are used to elicit information on the participant’s current situation and motivations. The goal is to help the FSW to see her situation clearly and accurately. Helping the individual to understand motivations that underlie her current unsafe behavior with clients is a prerequisite to changing her behavior^18^. In our pilot work, we learned that financial gain was the primary motivation for not using condoms with a client. It was not uncommon for FSWs to double their income if they were willing to have unprotected sex. Another common motivation for not using condoms focused on the type of client. Condom use was considered unnecessary if the client was elderly (“I’m not afraid of infection or pregnancy with him”) or a long-term client (“I like him and I trust him”). Other motivations underlying unsafe sex with clients included the lack of intimacy, discomfort, and bad taste associated with condoms. As the counselor and participant discuss these underlying motivations, the participant gains insight into her own behavior and she begins to build motivation for change. The information that emerges in the counseling session also provides the counselor with valuable information on the FSW’s values, expectancies, sense of self, and social/work context -- all of which influence motivation for change. Once awareness of the problem is achieved, the counselor will help the participant discover and understand her motivation for change. This is accomplished by spending time eliciting self-motivated reasons for change and enhancing the participant’s self-efficacy for change. In our pilot work, FSWs offered a plethora of self-motivational statements in relation to condom use with clients. FSWs motivations for using condoms with clients included the following: to avoid diseases (HIV & other STDS); to avoid pregnancy; to stay alive; to feel clean; to avoid direct contact with clients; to avoid getting sick again; to feel good about oneself; to avoid losing one’s job; to stay healthy for loved ones (family and children), to protect the client; to enjoy sex more; and to save time between clients. Counselors use the “decisional balance” approach^18^ to help the participant realize that, in most cases, reasons for using condoms with clients strongly outweigh reasons for not using condoms. Once the balance begins to shift in favor of positive change, the next step is to help the participant develop a plan of action that best suits her personal situation. The counselor asks about barriers to condom use. Barriers for female sex workers may include the threat of physical assault or death, rape, loss of clients, and loss of income. The counselor works with the participant to problem-solve these barriers. The counselor also offers information and suggestions on how to change behavior, and illustrates positive outcomes. The participant is actively involved in problem-solving and is encouraged to come up with solutions. The participant is presented with a menu of options. For example, the counselor presents a variety of safer sex options ranging from the use of male and female condoms for vaginal sex, to a variety of harm reduction strategies such as offering the client unprotected oral sex instead of unprotected vaginal sex. According to both motivational interviewing and social cognitive theory, belief in one’s ability to bring about change is an important motivator of change. It is very important that the participant attribute success to herself. The counselor also helps the participant to define achievable goals (e.g., always use a condom for vaginal sex with clients). Once the participant has defined goals and arrived at a plan of action, the participant and counselor engage in problem-solving exercises. Alternate choices of behavior and alternative strategies for dealing with the situation are discussed. Rewarding the participant with positive feedback helps to enhance self-efficacy. The counselor aims to strengthen the FSWs commitment to using condoms by exploring ways to make condom use exciting and erotic for the client. The counselor and participant also practice putting a condom on and taking it off a 5odríg penis model while discussing how to keep the man erect (e.g., licking penis shaft or testicles). The counselor and participant also discuss the participant’s successes, and the counselor makes suggestions for improvement if necessary. It was readily apparent in our pilot study that many FSWs did not know how to use a condom properly. The majority of women reported previous experiences with clients where a condom broke, tore, or stayed inside the vagina upon termination of the sexual act. For example, a common practice among sex workers in Tijuana is to use two condoms on the client’s penis. This practice resulted in condoms slipping off during sex or getting lodged in the vagina. Also, the popularity of long fingernails among FSWs in Tijuana contributed to frequent experiences with torn condoms. Condom failure also occurred as the result of using body lotions and vaginal medications as lubricant. Overall, FSWs in the pilot study expressed much gratitude for the condom demonstration exercise and the information regarding proper condom use. Demonstration and practice exercises are designed to enhance the participant’s attitudes, self efficacy, positive outcome expectancies, and intentions to use a condom. Creating a positive, supportive atmosphere that makes the use of condoms appealing to the individual is conducive to behavior change. The counseling session takes 40 to 45 minutes. The order and approximate duration of intervention activities are presented in the counselor training manual. They include: Assessing Readiness for Change (5-7 minutes); Decisional Balance Exercise (3 minutes); Building Motivation for Change (10 minutes); Identification of Problem & Problem-solving Exercise (7-10 minutes); and Knowledge & Skill-building Exercises (10 minutes).  DESCRIPTION OF STANDARD COUNSELING SESSION: The comparison condition for this research utilizes a modified version of the Centers for Disease Control’s revised guidelines for HIV counseling, testing, and referral^19^ and materials from Mexico’s National Center for AIDS Studies (CENSIDA). The face-to-face counseling session takes about 45 minutes. The focus of the counseling session is upon personal risk assessment and strategies for reducing personal risk. During the personal risk assessment component, the counselor helps the participant to identify, understand, and acknowledge behaviors and circumstances that put her at risk for contracting HIV and other STDs. The counselor also explores the participant’s previous attempts to reduce personal risk, and provides positive reinforcement for positive steps already taken. The counselor also helps the participant to set small, achievable risk-reduction goals and offers concrete suggestions for achieving personal goals. Basic educational information (e.g., HIV/STD transmission modes) is provided if the individual’s level of knowledge is low; however, the primary focus is on the discussion of transmission risk associated with specific behaviors or activities that are relevant to the participant’s personal risk. Lower risk alternatives to risk behavior are promoted (e.g., although oral sex is not risk-free, it is associated with lower risk for HIV infection as compared to unprotected vaginal sex).  MEASURES. All measures have been translated into Spanish and back-translated into English by a bilingual team of Latino/a researchers. In addition, all measured have been reviewed for cultural appropriateness by one of our collaborators, Dr. Nicolini, Director of the Mexican Psychiatric Institute in Mexico City.  BEHAVIORAL OUTCOMES. O’Leary et al.^20^ discuss the difficulties in selecting behavioral outcomes in HIV/STD prevention research; every measure has strengths and weaknesses. Behavioral outcomes in this study will include: frequency of unprotected vaginal, oral, and anal sex with clients and spouse/steady partner; protected sex ratio (number of protected sex acts divided by total number of acts); number of clients; number and type of other sex partners (non-clients); number of partners who inject; number and type of STDs; and incidence of HIV/AIDS.  Condom Use: We will assess condom use with clients and spouse/steady partner. FSWs will report the number of times they engaged in vaginal, oral, and anal sex without a condom (or dental dam) with clients and their spouse/steady partner during the past two weeks.  HIV Testing and Sexually Transmitted Diseases. In addition, participants will receive free screenings for HIV, syphilis, gonorrhea and Chlamydia, based on a blood sample and vaginal swab. Pre-test counseling will also be provided, which will include basic information on how these infections can be transmitted and which preventive behaviors can reduce risks. A trained phlebotomist will obtain a venous blood sample from each participant to permit antibody testing for HIV and syphilis. In the case of HIV, we will use a rapid antibody test which is considered highly sensitive and specific (called “Determine”, manufactured by Abbott). The test device, which resembles a dipstick, is then inserted into the vial. In as little as 20 minutes, the test device will indicate if HIV-1 antibodies are present by displaying two reddish-purple lines in a small window on the device. Depending on the results of the rapid HIV test, each participant will receive immediate post-test counseling before leaving the study site. In the event that a rapid test for HIV is positive, a second blood draw will occur immediately into a 10 cc CPT tube, which is necessary to extract viral RNA for genotypic analysis. We will also use this specimen confirm any positive results of the rapid HIV test using ELISA and confirmatory Western Blot, as a further quality assurance measure. As a result, the amount of blood for HIV-negative participants (approx. 2 tablespoons) will be somewhat less than for HIV-positive persons (4 tablespoons), but not significantly so. Persons testing HIV-positive on the rapid HIV test will be counseled that their test was positive and that, while it is highly likely that they are infected with the virus, we ask them to return in one month for confirmation based on the standard ELISA and Western blot test. In accordance with CDC guidelines, counseling includes education about risk reduction (e.g. abstaining from injection or sexual contact, or using sterile syringes or condoms).  The remainder of the blood sample will be shipped to a laboratory in San Diego County where an aliquot will be tested for syphilis antibody, and HIV antibody confirmation will be arranged for through the state laboratory in the case of a positive specimen. The second aliquot will be stored at -80C for future testing of STDs and blood borne pathogens including syphilis. Women will also be asked to provide two vaginal swabs which will also be shipped to the same laboratory. One swab will be prepared for ligase chain reaction to amplify genetic material for gonorrhea and Chlamydia. The other swab will be stored at -80C for future testing of STDs, if funding permits.  All women will be asked to return for test results within one month, at which time post-test counseling will occur. In the event that a participant tests positive for HIV or an STI, feedback will be provided by regular clinic staff (either a physician or psychologist) who are trained to provide this feedback in a sensitive manner in order to reduce psychological distress. Referrals to the Municipal Clinic in that city will be made where regular HIV treatment is provided.  Women will be compensated $30 U.S. per visit for their time. Although we are quite confident that these incentives will be effective, we will also utilize cohort maintenance procedures that were successfully implemented in our pilot study in Tijuana. These long-established principles which were delineated by Robins^15^, Boice^16^, and Showstack et al.^17^, have been modified for use with FSWs. Below, we describe some basic cohort maintenance procedures that were successful in our pilot study. Retention at six-month followup in the pilot study was 79 percent (21% attrition). In the proposed study, reasons for study attrition will be recorded for all participants, if known. Other STDs will be assessed by self-report (e.g., 6odríguez, syphilis). Self-report of STDs will be validated by a review of patient charts at each clinic. The review of patient charts will be conducted by the clinical coordinator at each site.  MECHANISMS OF CHANGE VARIABLES.  Knowledge: Our new measure of knowledge^21^ consists of 18 items, which assess the participant’s awareness of the importance of condom use with respect to HIV/STD prevention (e.g., “People who have been infected HIV quickly show serious signs of being infected”; “A person will not get HIV if she or he is taking antibiotics”). Response categories are True (1) or False (0). Internal consistency alphas range from .75 to .89, and test-retest reliability range from .76 to .94.  Self-Efficacy: Our 5-item measure of self-efficacy asks participants to indicate the extent to which they are able to use a condom properly with clients. Responses are coded on a 4-point scale (1=Strongly Disagree to 4=Strongly Agree). The alpha for this scale is .85.^22^  Outcome Expectancies: Participants respond to three items using a 4-point scale ranging from 1 (Strongly Disagree) to 4 (Strongly Agree). An example of a positive outcome expectancies is: “I believe that condoms will protect me from getting HIV). The alpha for this scale is .79.^22^  Attitudes toward AIDS Preventive Acts: Two items will be used to measure attitudes toward AIDS preventive acts^23^. “My not having (vaginal/oral/anal) intercourse with my clients during the next two months would be: very good (5), somewhat good (4), neither good nor bad (3), somewhat bad (2), and very bad (1)”; and “My always using condoms for (vaginal/oral/anal) intercourse with all my clients during the next two months would be (response categories noted above). Each item will be asked separately in relation to vaginal, oral, and anal sex. The alpha for this scale is .79.  Intentions to Engage in AIDS Preventive Behavior: Intentions will be captured by two items: 1) I intend not to have (vaginal/oral/anal) intercourse with my clients during the next two months; and 2) I intend to always use condoms for (vaginal/oral/anal) intercourse with all my clients during the next two months. Response categories are measured on a 5-point scale (5= very true; 4=somewhat true; 3=neither true nor untrue; 2=somewhat untrue; 1=very untrue)^23^. Each item will be asked separately in relation to vaginal, oral, and anal sex. The alpha for this scale is .89.  Perceived Social Norms: Perceived expectations for AIDS prevention behavior will be measured by two items: 1) Most people in my line of work think that I should not have vaginal intercourse with my clients during the next two months; and 2) Most people in my line of work think that I should always use condoms for vaginal intercourse with all my clients during the next two months. Response categories range from 1 (very untrue) to 5 (very true)^23^. These two items are repeated for oral sex and anal sex with clients. The alpha for this scale is .96.  CONTEXTUAL FACTORS  Working Conditions is a multidimensional construct with multiple indicators that include work site (e.g., brothel, street, bar), type of sex worker (e.g., dance hostess, street worker), degree of protection from drunk or aggressive clients, nature of relationship with pimp (if applicable), availability of condoms, earnings, type of sexual services performed, control over selection of clients. An overall rating of work conditions is also provided by the participant (1=very unfavorable) to (5= very favorable).  Drug and Alcohol Use will be measured by a 5-item scale developed by Temoshok & Nannis^24^. FSWs will be asked to report their use of alcohol, marijuana, cocaine, heroin, hallucinogens, and ecstasy preceding and during sex with clients for the previous two months. They will also be asked about their general use of alcohol and drugs. Frequency and amount consumed will be recorded at each assessment.  Victimization and Trauma will be measured by four items derived from the family/social relationships section of the Addiction Severity Index (ASI-F). The section concerning abusive relationships contains questions about past and present episodes of physical, emotional, and sexual abuse. Participants will be asked four questions in relation to male clients, pimp, and spouse/steady sex partner. Sample items include: “In the past 30 days, did any of your (male clients) abuse you emotionally (make you feel bad through harsh words, humiliation, manipulation; and “In the past 30 days did any of your (male clients) abuse you sexually (rape, forced sexual advances, or non-consensual sex acts)”. The ASI has good test-retest reliability (>.80), as well as concurrent and discriminant validity^25^.  SOCIAL FACTORS  Social Support will be assessed using a modified version of the Schaefer, Coyne & Lazarus Inventory^26^. Three dimensions of support will be assessed in relation to safer sex: (1) satisfaction with emotional support; (2) satisfaction with informational support; and (3) network support. The participant is provided with a list of 16 network members. She then rates each person on five items that tap the person’s reliability, trustworthiness, caring, confidant qualities, and information provided in relation to safer sex. A 5-point response scale is utilized (1=not at all to 5=extremely). Satisfaction with emotional and informational support is averaged across all network members. Network size is defined by the total number of persons rated. Internal consistency reliability for the original scale is .95; test-retest reliability is .66.  Social Influence assesses the extent to which specific social network members engage in high risk behaviors. Participants are presented with three categories of individuals: other female sex workers (4 FSWs with whom you have the most contact or feel closest to); pimp; and husband/steady partner. For each of these six individuals, the participant is asked to rated the extent to which this person engages in the following behaviors: sex with clients without condoms (N/A for pimp and steady); sex with multiple partners without condoms (other than clients for FSWs); drug use before or during sex; alcohol use before or during sex; and intravenous drug use. Ratings are made on a 4-point scale ranging from 1 (not at all) to 4 (very often).  Life Experiences Survey is a self-report measure that rates the desirability of positive and negative life experiences that have occurred during the past six months^27^. Endorsement of an item indicates that an event has occurred. Participants will be presented with a list of events or life changes that are typically experienced in the general population. The original list of 47 events was reviewed by a panel of experts from the Universidad de Baja Mexico and modified to enhance cultural sensitivity. Three items were dropped. Three blank spaces permit the participant to report and rate the occurrence of other events that do not appear on the list. Desirable life experiences and undesirable life experiences are rated separately using a 5-point scale ranging from –2 (extremely negative) to +2 (extremely positive). A positive change score is calculated by summing the ratings for life events designated as positive by the participant. Similarly, a negative change score is computed by summing the ratings for negative events or experiences. The positive and negative change scores will be summed to create a total change score, which represents all change experienced during the past six months. The original LES has acceptable internal consistency reliability and test-retest reliability, ranging from .54 to .88.  INTRAPERSONAL FACTORS.  Depressed mood will be assessed using the Beck Depression Inventory^28,29^. This measure consists of 21 items, each having four graded statements pertaining to how the participant has been feeling during the past week. The statements within a question are ordered (0 to 3) to showing increasing depressive symptoms. Summary scores are calculated (range 0 to 63). The items of the BDI are clinically derived and have undergone extensive reliability and validation studies. Internal consistency assessments of reliability have been high (>.9).  Self-esteem will be assessed using the Rosenberg self-esteem scale^30^. Self-esteem is an element of self-concept that refers to the regard in which one holds oneself. It is conceived as a global construct, the assessment of which is not tied to a particular context. The self-esteem scale consists of eight items that are measured on a 4-point scale ranging from 1 (Strongly Disagree) to 4 (Strongly Agree). Sample items include: “I feel that I have a number of good qualities”; “I wish I could have more respect for myself”; and “On the whole, I am satisfied with myself”. The self-esteem scale has good test-retest reliability, as well as construct validity.  BACKGROUND CHARACTERISTICS  Financial Need will be assessed by five items that ask about the participant’s earning from sex work, earnings from other sources of work, number of people who are financial dependents, and the nature of relationship with financial dependents. An overall rating of financial need is also obtained from participants.  INTERVENTION IMPLEMENTATION ISSUES.  Quality Assurance. We will utilize two CDC-endorsed strategies^31^ for ensuring counselor adherence to the protocol: 1) Supervisor Observations. The clinical coordinator in each treatment-comparison city will observe each counselor in session with a participant once per week for a period of three weeks post-training. Corrective feedback will be provided. Thereafter, supervisor observations will be conducted once every three months; 2) Participant Evaluations. This method involves asking each FSW to complete a brief checklist that queries the content of her counseling session.  Strategies to minimize self-report bias in sex research. We will utilize multiple strategies for minimizing self-report bias^32^: (1) We will emphasize confidentiality by assuring anonymity through the use of ID numbers, and strict rules regarding confidentiality; (2) Project staff will provide FSWs with assurance of confidentiality and reinforce the importance of providing honest responses; (3) Project staff will give participants an opportunity to discuss concerns or fears before and after enrollment; (4) To encourage participant “openness”, our project staff will be open, non-judgmental, and have good rapport-building skills; and (5) Project staff will be individuals who have previously worked at the clinic so that they are familiar faces to the FSWs.  Data System. The data system to support the coordination of this project must acquire, store and provide access to data associated with our protocol from our participating sites. We will continue to use the data management system developed as part of our pilot study. This includes weekly transfer of hard copies of data (note that no data were lost in this process), and weekly feedback in order to maintain data quality.  Quality Control of Data. Our research group has devised procedures to minimize data loss. All data are delivered to and tracked by the data manager in San Diego. All items are entered into a computer database with entry screens developed in SPSS-DE. These screens require double entry for data verification. Data are maintained and backed up daily to prevent loss and to ensure full retrieval for analyses. Security is maintained through a password system. Our database contains no personally identifiable data, and participant records are identified only by a sequential study identifier.  DATA ANALYSIS. All data analysis will be conducted at the UCSD Coordinating Center. The first stage of analysis will involve tests for normality of distributions for each variable, with appropriate transformations as required. We propose to utilize two general data analytic strategies: 1) Analysis of variance techniques (i.e., repeated measures MANCOVA and group x time repeated measures ANCOVA and MANOVA); and 2) multiple regression. |
| --- |

| **9. HUMAN SUBJECTS** |
| --- |

| The remainder of this project will be conducted in two primary medical clinics that are responsible for providing regular medical examinations to FSWs in Matamoros and Nuevo Laredo. The characteristics of our proposed sample are by design active FSWs. All participants will be Mexican Nationals residing in the two cities named. |
| --- |

| **10. RECRUITMENT** |
| --- |

| Recruitment Sources. Our remaining sample of 900 FSWs will be recruited through health clinics in Matamoros and Nuevo Laredo.  Participant Followup and Tracking. To enhance our understanding of the challenges associated with tracking a large cohort of FSWs in a third world country, Dr. Patterson contacted a number of international researchers with expertise in this type of research in a range of countries including Thailand, Africa, Dominican Republic, Vietnam, India, and Latin America. All experts agreed that the best strategy for assuring optimal followup is to conduct the intervention in a health clinic setting because FSWs will return for health care services. Other important suggestions for ensuring good followup included subject payment for participation, a non-coercive approach, and easy access to the clinic for FSWs from their place of work/residence. Since the proposed study will be conducted in municipal health clinics we anticipate (and it was our experience in the pilot study) that the women will be motivated to return to the clinic on a monthly basis as required by law in order to keep their health card (working permit) current.  1. The participant will be advised in her first contact that she will be followed for a six-month period. She will be told that she is expected to attend two sessions (i.e., assessment/counseling and followup session), and undergo a free gynecological exam and blood test at each visit.  2. The participant will also be asked at her first visit to provide us with the name and telephone number of a contact (girlfriend, fellow worker, bar manager) whom we may contact if we have difficulty locating her at six month follow-up.  At her first visit, the participant will be given a card with the counselor’s name and telephone number. On the back of the card, the counselor will write the date of the next visit and the amount of the payment.  4. In our pilot work, we learned that some FSWs will consent to project staff leaving word “on the street” in the event that they cannot be located for their six-month followup visit.  5. The Municipal Clinic offers flexible hours, which makes it easier for FSWs to make it in for their visit.  6. It is critical when working with this population to develop strong bonds of trust and confidence. To this end, project staff in each research site will be carefully chosen for their sensitivity, discretion, and understanding of the needs of the target population.  7. FSWs will be advised at their first visit that if they move away, they will provided with a bus ticket or voucher to get them back to the clinic for their followup visit (if they have moved 100 miles or less from the clinic site). |
| --- |

| **11. INFORMED CONSENT (Note: provide information in Section 28 on Surrogate Consent and Decisional Capacity Assessment, if applicable)** |
| --- |

| The informed consent procedure is designed to maximize the potential participant’s comprehension of study procedures and to ensure that participation is voluntary. Before a participant is enrolled in a study, the purpose, the procedures to be followed, and the risk and benefits of participation will be explained by the clinic staff (i.e., the interviewer). This process will be facilitated by a two-page bulleted summary of key points from the informed consent document. This is necessary based on recommendations from interviewers and counselors in the field who expressed concern with the complexity of the longer consent document. However, this does not preclude the interviewer from reading the complete document to the participant, but provides an additional opportunity to clearly inform the participant about the project. Since medical treatment staff will be supervised by our collaborators, we have detailed understanding of the setting and issues surrounding confidentiality. Consent forms have been written in simple language at a sixth-grade reading level. Details of study participation, including the need for follow-up contact, will be described in the consent form and explained verbally. The research staff who will be responsible for obtaining informed consent will assess whether the potential participant has understood the study and consent form by asking key questions (e.g., “How much time will this take you?”; “What are the possible benefits for you?”). After informing the participant both orally and with a written consent form, a signed informed consent from the study participant will be obtained. Prior to study implementation, we received approval from the UCSD Institutional Review Board (IRB) Committee on Human Research. Federal Certificates of Confidentiality allow us to protect participant information from outside requests and subpoenas in the U.S (approval already on record with IRB). The Universidad Autónoma de Tamaulipas, Comité de Bioética de la Facultad de Medicina de la Unidad Académica de Ciencias de la Salud y Tecnología (Bioethics Committee of the Faculty of Medicine and the Department of Health Sciences and Technology) has reviewed and approved the use of human subjects in study protocol .  Recruitment of subjects and informed consent procedures follow procedures used in our U.S.-based studies. Participants who are appropriate for inclusion will be approached by a staff member from this project who will describe the study. The description includes information on the questionnaire, time commitment, and content of the intervention. In all cases, fully informed consent will be obtained. Our Mexico-based collaborators will be directly available to answer any questions raised by a subject. Drs. Bucardo, Fraga, and Davila from our Mexico co-ordination center will also be available to address questions that may arise, and Dr. Patterson will be available for consultation with all collaborators. In addition, a copy of the consent form, which includes a description of the study, is provided to all subjects. |
| --- |

| **12. THERAPEUTIC ALTERNATIVES** (therapeutic studies only) |
| --- |

| N/A |
| --- |

| **13. POTENTIAL RISKS** |
| --- |

| Concerning interviews and the intervention, the main issue for the participant is the time taken. Further, since many of the questions and discussions are very personal, they could cause emotional distress. In addition, given the social networks surrounding prostitutes in Mexico, participants could experience physical or emotional violence if their partners or associates deem that the behavioral change suggested is not acceptable. Our interviewers are trained to allow subjects not to answer questions that seem to be distressing them or even to terminate an interview if it gets too stressful.  Concerning blood draws for HIV testing, there is a minimal risk of bruising at the site of venipuncture. As for the potential emotional impact of a positive test result, it should be noted that this population is accustomed to regular HIV and STD tests in a clinical setting. All staff are trained to provide counseling to minimize distress.  There is minimal risk of breach of confidentiality. This has not occurred, to our knowledge, in the thousand or so subjects that we have repeatedly interviewed regarding sensitive life events and other psychosocial interviews for which our group has been responsible. |
| --- |

| **14. RISK MANAGEMENT PROCEDURES** |
| --- |

| In addition to the risk management procedures already mentioned above, we have minimized the time needed to complete the interviews (without jeopardizing the quality of the information gathered) by thoroughly familiarizing the interviewers in the content of the questionnaires. As for confidentiality, the subject’s name is removed from the interview forms and an identification number is assigned. All references to or about a subject are made with these ID numbers. Further, all records, forms, and raw data are kept in secured file cabinets in our research office.  In the event that a study participant experiences significant problems, our Mexican co-investigators Drs. Fraga and Salazar will be contacted by clinic staff who are under their direction. Dr. Salazar is responsible for day-to-day operations in Matamoros and Nuevo Laredo, and he or his staff will be in direct contact with participants and will manage risks associated with interviews. A 1-800 number will be available for subjects to call.  We have obtained an NIH Certificate of Confidentiality which protects data when it is shipped to San Diego for data entry and analyses. There is no comparable legal document in Mexico. |
| --- |

| **15. POTENTIAL BENEFITS** |
| --- |

| Participation in this research project has a number of potential benefits to the individual. These include free HIV and STD testing and results. If results are positive referrals to treatment facilities will be made. Participants may learn about reasons to practice safer sex with their clients, personal motivations for doing so, strategies for negotiating safer sex with their clients, and information on how to use condoms. In addition, findings from this project may provide the basis for future intervention programs for Mexican sex workers. |
| --- |

| **16. RISK/BENEFIT RATIO** |
| --- |

| We judge that the benefits to participation in this project outweigh the riskswhich we will minimize wherever possible. Probably the most important risk associated with participation in this project is the risk that of a positive HIV test. This would result in the loss of the participant’s sex worker permit. However, on the positive side, the participant would be able to begin treatment and potentially prolong their life. In addition, the risk of unknowingly transmitting HIV to a client or other individual during a sexual contact would be averted. There are a number of other potential risks associated with participation in this project. These include the possibility that confidential information provided to research staff is inadvertently disclosed to someone outside this project. While this has never happened in the 22 years of the PI’s research experience, it is always possible. Therefore, we make every effort to guard against such happenings. This includes extensive training and monitoring of staff on issues of confidentiality, coding of data without names or other identifying data, and storage of data under the safest possible conditions. Other potential benefits for participation were outlined under Potential Benefits to participation. In our judgment, the potential benefits to the individual in the form of protecting their own health through learning about safer sex practices, information about their own health with associated treatment referrals, and the potential knowledge that this study will provide to science and public health officials outweigh the potential risks associated with participation in this project. |
| --- |

| **17. EXPENSE TO SUBJECT** |
| --- |

| None other than time taken. |
| --- |

| **18. PAYMENT FOR PARTICIPATION** |
| --- |

| Participants will be paid $30.00 per visit (maximum of two visits) for participation in this project. They will also be compensated indirectly in that their laboratory tests (i.e., for STD/HIV) will be paid for by this study, and they will be given approximately 100 condoms. |
| --- |

| **19. PRIVILEGES/CERTIFICATIONS AND LICENSES** |
| --- |

| Thomas L. Patterson, Ph.D. is a Professor In Residence, Department of Psychiatry, UCSD School of Medicine, is the Principal Investigator for this project and is responsible for overseeing all its aspects.  Jesus Bucardo, M.D., Clinical Instructor, Department of Psychiatry, UCSD School of Medicine. Dr. Bucardo is a Mexican born physician in clinical practice in San Diego, CA. He received his medical training in Mexico. He then obtained an MPH at SDSU and a postdoctoral fellowship in epidemiology at the UCSF Center for AIDS Prevention Studies (CAPS). He completed his residency in psychiatry at UCSD and worked as a clinician/researcher at the HNRC. Dr. Bucardo is knowledgeable as to the social, cultural, and political aspects of conducting HIV/STD prevention work in Mexico. He conducted HIV/STD seroprevalence studies in Baja, Mexico in the 1990s and has been instrumental in terms of implementing and coordinating the Tijuana-based pilot project. In the proposed study, Dr. Bucardo will coordinate and facilitate communication between the Mexico and San Diego-based Coordinating Centers. He will also help to train counselors at intervention sites, monitor counselors, respond to their problems and concerns, and liaison with administrators at the municipal health clinics at various intervention sites. Dr. Bucardo will also participate in regular meetings with Drs. Patterson and Fraga, assist with the planning and conducting of data analyses, and assist in the writing of progress reports and manuscripts for publication.  Wendy Davila, M.D., University Center for Medical Assistance and Investigation, Tijuana, Baja California. Dr. Davila trained counselors for the pilot project for this study, and supervised counselors during the study. Her responsibilities during the present project will be to train counselors and provide ongoing feedback and retraining as needed.  Adela de la Torre, Ph.D., Director & Professor Chicana/o Studies Program, and Adjunct Professor* Family and Community Medicine at University of California, Davis, will be responsible for the overall administration and coordination of the University of California Davis Research Team as well as assist Dr. Patterson with Dr Fraga’s Mexican team in grant implementation activities. Dr. de la Torre’s research on Latina/o health issues has resulted in her placement on key national health advisory boards such as the National Advisory Council of the Center for Substance Abuse Prevention (CSAP/SAMHSA) and to be recognized by the U.S. Surgeon General for her participation in the Surgeon General’s Hispanic/Latino Health Initiative. Dr. de la Torre has five years of experience directing binational programs as Director of the Border Academy (five years at the University of Arizona and one year at UC Davis), a program that focuses on educating Mexican and US health professionals on border health issues. Her research expertise in border health issues will provide cultural sensitivity to the unique regional issues in implementation of this intervention program. Finally, Dr. de la Torre, with her research team will assist Dr. Patterson in the interpretation, analysis and final publication of reports and manuscripts related to this grant.  Yvette Flores Ortiz, Ph.D. an Associate Professor Chicana/o Studies at UC Davis. Dr. Ortiz will work with Dr. de la Torre and Dr. Garcia in monitoring program implementation goals at the clinical sites to ensure data integrity. In addition, given her considerable clinical experience in practice with high risk Latina women, she will play a central role in developing the cultural and linguistically sensitive training protocol for all staff and interventionists involved with FSWs. In addition, she will play a central role in analyzing the assessment instruments used for developing the appropriate cultural and gender cues selected for the safer sex intervention for FSWs. Dr Flores-Ortiz will work closely with the Mexican research and clinic teams in developing the appropriate culturally sensitive training protocols for the interventionists in the clinics. Finally, along with Dr. de la Torre and Dr. Garcia, she will assist in interpreting the results of the survey as well as play an active role in publication with other members of the research team. This will include scheduled research reports and peer reviewed articles.  Miguel Fraga, M.D., Professor of Public Health, Universidad Autonoma de Baja California is the Principal Investigator, Mexico, will lead the Mexico-based team. Dr. Fraga holds a specialty in community medicine and a Master’s degree in Medical Education. He is Professor of Medicine and Public Health, at the UABC-Tijuana School of Medicine. He was the Mexico-based principal investigator in the Tijuana –COMPARTE SEXO MAS SEGURO– Pilot Study. As the leader of the Mexican-based research team, Dr. Fraga will be responsible for directing the UABC-based Coordinating Center and supervising the UABC team. He will also coordinate work of the four target cities and serve as liaison between the UCSD and the UABC research teams. Dr. Fraga will meet with Drs. Patterson and Bucardo on a regular basis to discuss research issues and address the progress and direction of the project. Dr. Fraga will be responsible for coordination of staff at all four study sites. In addition, he will help to train and supervise the intervention counselors in all study sites and monitor the scientific rigor required in a multi-site intervention. Dr. Fraga will oversee data collection at the multiple study sites, monitor quality assurance of data packages, and ensure the integrity of the data.  Lorena Garcia, MPH, Dr. PH., Assistant Professor Chicana/o Studies at UCD, will serve as research coordinator for the UC Davis team. She has played a lead role in checking the translations of all instruments and consents for this project. Dr. Garcia will report directly to Dr. de la Torre and will assist her in reporting responsibilities associated with the award. She will also supervise the work of graduate students involved with the UCD research training center. Dr. Garcia is well versed in epidemiological research on Latina health, particularly with respect to the Mexican origin population in the US and in Mexico. Dr. Garcia brings to the grant her vast research coordinator experience with Latino research projects in the following areas: health insurance status of Latinas, inhalant abuse among Latino youth, community based prevention/intervention programs to prevent HIV infection in pregnant women at high risk, community-based health initiatives to address Latino communities and ethnic and cultural difference in health behavior and health problems, injury, acculturation and intimate partner violence among Latinas.  Esmeralda Iniguez, MPH – PhD Student Trainee. Ms. Iniguez is a degree candidate in the UCSD/SDSU Joint Doctoral Program in Epidemiology. For her dissertation she will conduct secondary analysis of this project’s data. No participant identifiers will be used as part of this analysis, only aggregated prevalence and risk behavior data. Ms. Iniguez will use mathematical modeling techniques to develop the best models to predict the course of the HIV epidemic for female sex worker (FSWs) in Tijuana over the next 30 years. Simulations will be generated to forecast the potential impact of expanded access to VCT, condoms and sterile syringes.  Sandra Larios, M.S. – PhD Student Trainee, is a degree candidate in the UCSD/SDSU Joint Doctoral Program in Clinical Psychology. For her dissertation, Ms. Larios proposes to use data gathered from the first phase of our study (Tijuana and Ciudad Juárez) to examine the applicability of the Social Ecological model for HIV prevention. Data will be used to examine how individual level factors (self efficacy, condom use beliefs, and substance use before sex) mediate the relationship between latent constructs (interpersonal, institutional, community, and policy) and condom use in FSWs. Multiple group structural equation modeling will be performed comparing women who work on the street (N=462) to those who work in a bar or brothel (N=333) in order to help develop more effective HIV prevention interventions for this population.  Carlos Magis Rodríguez, M.D., Director of Epidemiology and Research, Central para el Control y Prevención de SIDA (CENSIDA). Dr. Magis will be our Consultant on HIV/AIDS Epidemiology and Research in Mexico. He will contribute expertise during the initial planning phase on issues of HIV/AIDS in Mexico. He will provide advice to guide the development of the project in accordance with the policy and protocols of the federal government of Mexico. This will enhance the feasibility of transferring the model developed through this study for application at the national level.  Prisciliana Quijada Orozovich, MPH, continues to serve as the Project Manager and Data Coordinator. She is responsible for supervising and training of other professional counselors, staff personnel and student aides; planning & assigning work tasks; managing all aspects of research trials; creating methods for maintaining all study-related documents; supervising database management; supervising administration of skills training protocols, structured interviews and screening patients; developing and maintaining study manuals; preparing human subjects protocols; making original contributions to publications, research design and grant writing. She also supervises and perform a wide variety of field recruitment and networking tasks.  Lic. Carlos Reyna, Attorney at Law, Professor of Law, UABC Tijuana School of Law. Mr. Carlos Reyna is a professor of law and an attorney at the UABC-Tijuana School of Law. He will serve as legal counsel for our project and will be available to help as laws and regulations change and avoid potential legal problems in this sensitive arena in Mexico.  Juan Salazar Reyna, M.D. Coordinator of Medical Training, Instituto de Ciencias Biomédicas, Universidad Autónoma de Matamoros. Each of our intervention and comparison sites will have a site manager (a physician) who has an appointment at the nearest Mexican University. Dr. Juan Salazar will take on the role of site manager for Matamoros. The site manager will be responsible for the overall scientific direction and management of the intervention at the designated clinic. He will coordinate with the Mexico Training Coordinator on a daily basis, and will be responsible for accessing the clinic population, ensuring adherence to the study protocol and the timely collection of data, and for the training and overall direction of study personnel.  Lawrence Schneiderman, M.D., Professor, Family and Preventive Medicine, UCSD School of Medicine. Dr. Schneiderman is a Professor in the Department of Family and Preventive Medicine at UCSD with specific expertise in bioethical issues including those of informed consent, and has been active in teaching and research on ethical issues in medicine and psychiatry for several decades. Dr. Schneiderman has been PI or co-investigator in several studies related to ethics and informed consent. He also serves as a member of the Ethics Committee at the UCSD Medical Center. He is a nationally recognized medical ethicist with several publications in this field. Dr. Schneiderman will provide scientific consultation and guidance for this project including design, implementation and analysis of study results. Dr. Schneiderman will also participate actively in publications and presentations related to this project.  Shirley J. Semple, Ph.D., Staff Research Associate, Department of Psychiatry, UCSD School of Medicine. Dr. Semple has been working with Drs. Patterson and Grant at the HNRC for the past ten years. She is currently a co-investigator on Dr. Patterson’s NIMH-funded behavioral intervention to reduce high risk sexual behaviors of “at risk” HIV-negative, heterosexually-identified men and women, and the NIDA-funded intervention for HIV+ meth-using Men Who Have Sex with Men. She helped to develop intervention materials, trained intervention personnel, and oversees day-to-day activities on the currently-funded projects. Dr. Semple has also been instrumental in the development and testing of intervention materials for the proposed study of FSWs. She also helped to train the Tijuana-based researchers in motivational interviewing and served as the U.S. data manager and data analyst for the pilot project. In the proposed study, Dr. Semple will serve as the UCSD onsite investigator. She will participate in counselor training, supervise the project staff in San Diego on a day -to-day basis, ensure the accuracy of data entry, collaborate on planning analyses, conduct data analyses, assist in the writing of progress reports, and prepare articles for publication. Dr. Semple will also work closely with Dr. Bucardo to ensure that the English version of intervention materials are continuously updated, and that documentation of changes are recorded.  Hugo Salvador Staines Orozco, M.D., Coordinador of Medical Training, Instituto de Ciencias Biomédicas Universidad Autónoma de Ciudad Juárez. Dr. Hugo Staines is the site manager for Ciudad Juarez and has been responsible for the overall scientific direction and management of the intervention at the designated clinic. He will continue to be involved in the study in data evaluation, consulting, and the preparation of manuscripts.  Steffanie Strathdee, Ph.D., Harold Simon Chair of International and Cross Cultural Medicine, Department of Family and Preventive Medicine, UCSD, School of Medicine. Dr Strathdee is an epidemiologist who specializes in infectious diseases. Her role on this project will be to oversee the collection, processing, and interpretation of results from STI/HIV tests. She will also participate in data analyses and the development of manuscripts.  Monica D. Ulibarri, Ph.D., has applied for a minority supplement to the NIMH grant that funds this study. If funded, Dr. Ulibarri will conduct a sub-study testing a path model of hypothesized relationships between childhood sexual abuse, depression, relationship power imbalances, substance use, and HIV risk with a Supervisor of the San Diego State University Psychology Clinic. Dr. Ulibarri received her Ph.D. in 2005 from Arizona State University. Her dissertation was “Latina Women’s HIV Risk Study: The Relationship between Childhood Sexual Abuse, Mental Health, and HIV Risk.” Dr. Ulibarri has met repeatedly with Dr. Patterson to build and refine the design for her sub-study, which will involve a short, additional questionnaire to be administered by existing project staff at the Matamoros and Nuevo Laredo sites. Dr. Ulibarri will then handle and analyze the data in according to our overall confidentiality policies and develop manuscripts to publish her findings.  Oralia Loza, M.A. - PhD Student Trainee. Ms. Loza is a doctoral student in the UCSD/SDSU Joint Doctoral Program in Epidemiology.  For her dissertation, she will conduct secondary analysis of this project's data. No participant identifiers will be used as part of this analysis. Demographic and behavioral measures (i.e. drug use), history of abuse and migration, as well as working conditions and lab data (i.e. HIV and STI results) will be used in the analyses. Ms. Loza will perform statistical analysis on these baseline measures collected in two Mexico-US border cities (Tijuana, BC and Ciudad Juarez, CHI) among female sex workers to determine (1) correlates of early initiation into sex work, (2) behaviors independently associated with STI, and (3) the temporal association between drug use and sex work. |
| --- |

| **20. BIBLIOGRAPHY** |
| --- |
| 1. Secretaria de Salud y Asistencia (SSA), Instituto Nacional de Salud Publica, Consejo Nacional para la Prevencion y Control del Sida (CONASIDA). (2001). Reporte Anual de VIAH/SIDA –2000, Mexico, D.F., Mayo 2001.  2. Instituto Nacional de Estadistica y Geografia e Informatica (INEGI). Resultados Preliminares del XII Censo Nacional de Poblacion y Vivienda-2000 [Preliminary Results for the XII National Population and Housing Census-2000]. Press Release, June 21, 2000. Mexico City  3. [not used]  4. [not used]  5. Carrier, J.M. (1985). Mexican male bisexuality: A cross-cultural perspective. Journal of Homosexuality, 11, 75-85.  6. Secretaria de Salud y Asistencia (SSA), Instituto Nacional de Salud Publica, Consejo Nacional para la Prevencion y Control del Sida (CONASIDA). (1997). Reporte Anual de VIAH/SIDA –2000, Mexico, D.F., Mayo 1998.  7. Hyams, K.C., Escamilla, J., Lozada, R., Macareno, A., Bonilla, G., Papdimos, T., Martinez, C., & Gonzalez, P. (1990). Hepatitis B infection in a non-drug abusing prostitute population in Mexico. Scandinavian Journal of Infectious Diseases, 22(5), 527-531.  8. Guerena-Burgueno, F., Beneson, A.S., & Sepulveda-Amor, J. (1991). HIV-1 prevalence in selected Tijuana sub-populations. American Journal of Public Health, 81(5), 623-625.  9. Guerena-Burgueno, F., Beneson, A.S., Sepulvada-Amore, J., Ascher, M.S., Vugia, D.J., & Gallo, D. (1992). Prevalence of human T cell lymphotropic virus types 1 and 2 (HTLV-1/2) in selected Tijuana subpopulations. American Journal of Trop Medicine Hygiene, 47(2), 127-132.  10. Valdespino, J.L.; Garcia, M.L.; Del Rio, A.; Loo, E.; Magis, C.; Salcedo, R.A. (1995). Epidemiologia del SIDA/VIH en Mexico. Salud Publica de Mexico. 37(6):520-524  10a. Patterson, T.L., Semple, S.J., Fraga, M., Bucardo, J., De la Torre, A., Salazar, J., Orozovich, P., Staines, H., Amaro, H., Magis, C., and Strathdee, S.A. Comparison of sexual and drug use behaviors between female sex workers in Tijuana and Ciudad Juarez, Mexico. Substance Use & Misuse. In Press.  10b. Patterson, T.L., Fraga, M., Staines, H., Bucardo, J., De la Torre, A., Salazar, J., Semple, S.J., Orozovich, P., Pu, M., Amaro, H., Magis-Rodríguez, C., and Strathdee, S.A. High prevalence of HIV and sexually transmitted infections among female sex workers associated with injection drug use in two Mexico-U.S. border cities. Poster No. TUPE0283 presented at the XVI International AIDS Conference, 13-18 August, 2006, Toronto, Canada.  10c. De la Torre, A., Garcia, L., Flores, Y., Semple, S.J., Bucardo, J., Fraga, M., Staines, H., Amaro, H., Magis-Rodríguez, C., Strathdee, S.A., and Patterson, T.L. The impact of price and workplace on female sex worker condom use in Tijuana and Ciudad Juarez. Prevention and Intervention in the Community, Special Issue on Women and International HIV Prevention. Submitted June 2006.  10d. Larios, S.E., Semple, S.J., Fraga, M., Bucardo, J., de la Torre, A., Salázar, J., Orozovich, P., Staines, H., Amaro, H., Magis-Rodríguez, C., Strathdee, S.A., and Patterson, T.L. Risky business: A comparison of risk behaviors among female sex workers who work in bars versus the street in two Mexican-U.S. border cities. Poster presented at the XVI International AIDS Conference, Toronto, Canada, August 13–18, 2006.  11. Personal Correspondence. Health officials in Tijuana (July, 2001).  12. Secretaria de Turismo (SECTUR), (2000) Gobierno Federal de Mexico, Reporte: Actividad Fronteriza, publicado por la Secretaria de Turismo-Julio, 2000.  13. California Department of Health Services, Office of AIDS. (1993, October/November). HIV Education & Prevention Services Bulletin, 1(2).  14. DiClemente, R.J., & Peterson, J.L. (1994). Changing HIV/AIDS risk behaviors. The role of behavioral interventions. In R.J. DiClemente & J.L. Peterson (Eds.), Preventing AIDS: Theories and Methods of Behavioral Interventions (pp.1-4). New York: Plenum Press.  15. Robins, L.N. (1969). Deviant children grow up. St. Louis: C.V. Mosby.  16. Boice, J. (1978). Followup methods to trace women treated for pulmonary tuberculosis. American Journal of Epidemiology, 107, 127-139.  17. Showstack, J., Hargraeves, W., & Glick, L. (1978). Psychiatric followup studies: Practical procedures and ethical concerns. Journal of Nervous and Mental Disease, 166, 34-43.  18. Miller, W.R., & Rollnick, S. (1991). Motivational Interviewing: Preparing people to change addictive behavior. New York: The Guilford Press.  19. Centers for Disease Control and Prevention (2001). Revised guidelines for HIV counseling, testing, and referral. Atlanta: CDC.  20. O'Leary, A., DiClemente, R., & Aral, S. (1997). Reflections on the design and reporting of STD/HIV behavioral intervention research. AIDS Education and Prevention, 9, Supplement A, 1-14.  21. Carey, M.P., & Schroder, K.E. (in press). Development and psychometric evaluation of the brief HIV knowledge questionnaire (HIV-KQ-18). AIDS and Behavior.  22. Semple, S.J., Patterson, T.L., & Grant, I. (2002). Gender differences in the sexual risk practices of HIV+ heterosexual men and women. AIDS and Behavior, 6(1), 45-54.  23. Fisher, J.D., Kimble-Willcutts, D.L., Misovich, S.J., & Weinstein, B. (1998). Dynamics of sexual risk behavior in HIV-infected men who have sex with men. AIDS and Behavior, 2(2), 101-113.  24. Temoshok, L., & Nannis, E.D. (1992). Tri-Service Biopsychosocial Study protocol. The Military Medical Consortium for Applied Retroviral Research, Rockville, MD.  25. McLellan, A. T., Kushner, H., Metzger, D., Peters, R., Smith, I., Grisson, G., Pettinati, H., & Argeriou, M. (1992). The fifth edition of the Addiction Severity Index. Journal of Substance Abuse Treatment, 9, 199-213.  26. Schaefer, C., Coyne, J.C., & Lazarus, R. (1981). The health-related functions of social support. Journal of Behavioral Medicine, 4, 381-406.  27. Sarason, I., Johnson, J., & Siegel, J. (1978). Assessing the impact of life changes: Development of the Life Experiences Survey. Journal of Consulting and Clinical Psychology, 46(5), 932-946.  28. Beck, A.T. (1967). Depression: Clinical, Experimental and Theoretical Aspects. New York: Harper & Row.  29. Beck, A.T. (1976). Cognitive Therapy and Emotional Disorder. New York: Hoeber.  30. Rosenberg, M. (1965). Society and the adolescent self-image. Princeton, NJ: Princeton University Press.  31. Kamb, M., Dillon, B., Fishbein, M., Willis, K., & the project RESPECT study group (1996). Quality assurance of HIV prevention counseling in a multi-center randomized controlled trial. Public Health Reports, 111 (Supp.), 70, 503-509.  32. Robins, A.G., Dew, M.A., Lawrence, A.K., & Becker, T. (1997). Do homosexual and bisexual men who place others at potential risk for HIV have unique psychosocial profiles? AIDS Education and Prevention, 9(3), 239-251. |
|  |

| **21. INDUSTRY STUDIES** |
| --- |

| Not applicable |
| --- |

| **22. FUNDING SUPPORT FOR THIS STUDY** |
| --- |

| NIH 5 R01 MH065849-04 |
| --- |

| **23. BIOLOGICAL MATERIALS TRANSFER AGREEMENT** |
| --- |

| Not applicable. |
| --- |

| **24. INVESTIGATIONAL DRUG FACT SHEET** |
| --- |

| Not applicable. |
| --- |

| **25. IMPACT ON NURSING STAFF** |
| --- |

| There is no impact on nursing staff. |
| --- |

| **26. CONFLICT OF INTEREST** |
| --- |

| Not applicable. |
| --- |

| **27. CANCER-RELATED STUDIES** |
| --- |

| Not applicable. |
| --- |

| **28. PROCEDURES FOR SURROGATE CONSENT AND/OR DECISIONAL CAPACITY ASSESSMENT** |
| --- |

| Not applicable. |
| --- |

Version date 3/30/2004
